# Mapping genes for resilient dairy cows by means of across-breed genome-wide association analysis

**DOI:** 10.1186/s12864-025-11940-z

**Published:** 2025-10-01

**Authors:** Franziska Keßler, Maximilian Zölch, Robin Wellman, Jörn Bennewitz

**Affiliations:** Institute of Animal Science, University of Hohenheim, Garbenstr. 17, 70599 Stuttgart, Germany

**Keywords:** Resilience, Variance, Autocorrelation, Linkage Disequilibrium, GWAS, MDS, Dairy cattle

## Abstract

**Background:**

Indicator traits based on variance and autocorrelation of longitudinal data are increasingly used to measure resilience in animal breeding. While these traits show promising heritability and can be routinely collected, their genetic architecture remains poorly understood. We conducted GWAS for three resilience indicators across German Holstein (*n* = 2,300), Fleckvieh (*n* = 2,330), and Brown Swiss (*n* = 1,073) dairy cattle (*Bos Taurus*) populations. The indicators included variance (${v}_{d}$) and autocorrelation (${r}_{Auto}$) of deviations of observed from predicted daily milk yield and variance of relative daily milk yield (${v}_{r}$). Additionally, we analysed a selection index combining these traits. Prior to GWAS, we examined population structure through multi-dimensional scaling (MDS) and LD patterns, revealing distinct genetic clusters for each breed and similar LD decay patterns.

**Results:**

The GWAS results confirmed the polygenic nature of resilience, with multiple genomic regions showing significant associations. Notable signals were detected on *BTA5* (${v}_{r}$), *BTA14* (${v}_{d}$), *BTA2* and *BTA8* (${r}_{Auto}$) for single indicator traits. For selection index resilience, strong suggestive SNPs are located on *BTA4*, *BTA16*, *BTA21*, and *BTA27*. Detected regions overlapped with previously reported QTLs for performance, reproduction, longevity and health, providing new insights into the biological pathways underlying dairy cattle resilience.

**Conclusions:**

Our findings demonstrate that resilience indicators have a complex genetic architecture with both breed-specific and shared components, supporting their potential use in selective breeding programs while highlighting the importance of careful trait definition.

**Supplementary Information:**

The online version contains supplementary material available at 10.1186/s12864-025-11940-z.

## Background

In a challenging environment with an increased incidence of external disturbances such as heat waves intensified by climate change [1], there is a growing need for performance-stable and healthy livestock. One option is to breed for resilience, i.e., for the ability of an individual to react to short-term disturbances and maintain or quickly restore its own homeostasis and performance level [2, 3].

Since directly measuring responses to each disturbance is impractical in commercial settings, indirect approaches using routinely collected longitudinal data have been developed. The most promising is the use of indicator traits to determine performance variability and recovery speed. Performance variability can be expressed by the fluctuation of daily milk yield. Therefore, the variance of deviations between observed and predicted daily milk yield (${v}_{d}$) is calculated. The variance is small for cows with smooth lactation curves, but also for cows with low milk yield [2, 4, 5]. To addresses the influence of the milk production level, a second parameter is the variance of relative daily milk yield (${v}_{r}$)[6]. This parameter is small for cows with smooth lactation curves that show a low decrease in milk yield during the lactation. The heritability for both variance-based indicator traits are moderate, in the range of 0.13 to 0.24 [4–6]. Furthermore, the genetic correlations with performance traits are desirable when the standardized ${v}_{r}$ is used or ${v}_{d}$ is corrected for the original performance level [5, 6]. Several studies have shown that resilient individuals have better values for health traits [5, 7, 8] or the productive lifetime [4, 9] than non-resilient cows. Recovery speed is captured by the autocorrelation (${r}_{Auto}$) calculated from the deviations between observed and predicted daily milk yield of a cow over the time of one lactation. A resilient cow with fast recovery has a value for ${r}_{Auto}$ near to 0 [2]. The heritability is low with an average of 0.05 across several studies and breeds, and genetic correlations with other traits were mostly desirable but rarely significant [5, 8, 10]. A selection index can combine different resilience indicators in an optimal way. Since the economic value of resilience is difficult to measure, it was not used to determine the weighting factors of the single indicator traits and alternative approaches were considered. Previous studies offer two options: On the one hand, the genetic correlation between resilience indicators and health and functional traits could be considered [11], on the other hand, the weighting factors could be optimized by maximizing the selection response in health traits when breeding for resilience [8]. The latter is used in the following.

While previous studies estimated heritabilities and genetic correlations [4–6], and discussed the power and accuracy of single resilience indicator traits [2, 12], deeper analysis of the genetic architecture are lacking. To our knowledge, there has been one GWAS of resilience indicators in dairy cows [13], pigs [14] and laying hens [15]. Their results indicate a polygenic nature of resilience, with many gene loci having a small effect. Chen et al. [13] detected a strong peak on *BTA14* for performance variability.

This study builds on this state of research and uses in-depth genomic analysis to analyze the genetic architecture and relationships to other traits. Therefore, the aim of this study was to assess the genetic architecture of several resilience indicator traits by GWAS in the three large German dairy cattle (*Bos Taurus*) breeds German Holstein (HF), German Fleckvieh (FV) and German Brown Swiss (BS) using imputed HD SNP genotypes. Since we performed an across-breed and a within-breed GWAS, we also studied the across- and within-breed LD pattern and the population structure. This provided us information on the genetic relationship between the three breeds and supports the interpretation of the GWAS results.

## Methods

Data processing and evaluation as well as statistical analyses were performed in software R 4.2.1 [16]. Genotype preparation, phasing, imputation and population analysis were done by using PLINK 1.9 and PLINK 2.0 [17] and Beagle 5.4 [18, 19]. ASReml-R 4.1.0 [20] was used for univariate estimation of breeding values and GCTA 1.94.1 [21] for GWAS with de-regressed GEBVs as phenotypes.

### Materials

The dataset consists of 5,703 dairy cows of the breeds German Holstein (HF, $n=$ 2,300), German Fleckvieh (FV, $n=$ 2,330) and German Brown Swiss (BS, $n=$ 1,073), originating from 48 farms. The farms are members of the breeding organization Rinderunion Baden-Württemberg e.V. (Herbertingen, Germany) and work with automatic milking systems. The milking data, consisting of yield per milking and start time of milking, from the automatic milking systems were longitudinally recorded between October 2017 and April 2024 and were provided by the Landeskontrollverband Baden-Württemberg e.V. (Stuttgart, Germany).

Animals in our study were genotyped with 50 K SNP chips. For HF, the Eurogenomics-MD-Chip was used in several versions. The merging of the chips and the imputation of undetermined SNPs were done by Vereinigte Informationssysteme Tierhaltung w.V. (Verden, Germany). Genotyping of FV and BS was done by Landeskontrollverband Bayern (Munich, Germany) and Bayerische Landesanstalt für Landwirtschaft (Grub, Germany) using Illumina BovineSNP50v2 BeadChip or DAC-BS50 Illumina. For both breeds, we obtained a standardized data set over the different chips, whereby missing SNPs were called with the population mean. We excluded SNPs on sex chromosomes, on undefined position, with minor allele frequency (MAF) < 0.03 or missing call rate > 0.01. The MAF threshold was stricter than in comparable studies [13, 22] in order to cause fewer false-positive results in the GWAS and was based on a study with a similar data set to our BS sample [23]. SNPs that are not included in the high-density reference panels were excluded, as they would be automatically removed during imputation. All genotypes were updated to the reference genome assembly ARS-UCD1.2 by manually replacing the existing coordinates with the updated ones [24]. SNPs, which are not listed in the ARS-UCD1.2, were excluded. This left 31,416 SNPs for HF, 34,238 SNPs for FV and 31,527 SNPs for BS.

A detailed overview of the size of the raw genotype and phenotype data and remaining data sets after filtering per breed can be found in the Supplementary (see Additional File 1).

### Methods

#### Calculation of resilience indicator traits

Milk yields per milking were added up to daily milk yields, whereby the first milking was divided proportionally between the current and the previous day. Daily milk yields were filtered as follows. Days where the number of animals milked on the farm deviated by three standard deviations from the mean number of animals normally milked on the farm as well as the day before and after were removed for all animals on this farm. Furthermore, the day before and after an individual gap of an animal, meaning one or more days without available data, was removed. Lactations were considered from day 10 to day 305, and those with less than 50% coverage respectively 148 data points were excluded. For each lactation of each cow, a lactation curve was predicted using spline interpolation with *pspline* in R [25] with degrees of freedom set to 5 and data points weighted by the strength of assumed disturbances. Details are described in Keßler et al. [6].

We considered three resilience indicator traits. The variance of the deviations between observed and predicted absolute daily milk yield (${v}_{d}$) was calculated as:

$${v}_{d}\left({\mathbf{y}}_{il}\right)=\frac{1}{n-1}*\sum_{t=10}^{n}{\left(\left({{\varvec{y}}}_{{\varvec{i}}{\varvec{l}}{\varvec{t}}}-{\widehat{{\varvec{y}}}}_{{\varvec{i}}{\varvec{l}}{\varvec{t}}}\right)-\overline{\left({{\varvec{y}}}_{{\varvec{i}}{\varvec{l}}}-{\widehat{{\varvec{y}}}}_{{\varvec{i}}{\varvec{l}}}\right)}\right)}^{2}$$

with ${\varvec{y}}$ a $n$-vector with daily milk yields of a cow $i$ in a certain lactation $l$, $\widehat{\mathbf{y}}$ the vector with interpolated daily milk yields and $t$ the lactation day. The variance of relative daily milk yields (${v}_{r}$) in considered lactation period was calculated as:

$${v}_{r}\left({\mathbf{y}}_{il}\right)=\frac{1}{n-1}*\sum_{t=10}^{n}{\left(\left({\alpha }_{ilt} {\mathbf{y}}_{ilt}\right)-\overline{\left({\alpha }_{il} {\mathbf{y}}_{il}\right)}\right)}^{2}$$

with ${\varvec{y}}$ a $n$-vector with daily milk yields of a cow $i$ in a certain lactation $l$ and the scaling factor ${\alpha }_{il}=\frac{100}{\sum_{t=10}^{n}{y}_{ilt}}$ with $t$ the lactation day. The two parameters were logarithmized to conform to the assumption of normal distribution according to previous analyses in Keßler et al. [6].

The autocorrelation of the deviations between observed and predicted absolute daily milk yield (${r}_{Auto}$) was calculated as:

$${r}_{Auto}\left({\mathbf{y}}_{il}\right)=\frac{cov\left({\mathbf{y}}_{il}-{\widehat{\mathbf{y}}}_{il}\right)}{var\left({\mathbf{y}}_{il}-{\widehat{\mathbf{y}}}_{il}\right)}$$

and in detail:

$${r}_{Auto}\left({\mathbf{y}}_{il}\right)=\frac{\frac{1}{n-2}*\sum_{t=2}^{n}\left(\left(\left({{\varvec{y}}}_{{\varvec{i}}{\varvec{l}}{\varvec{t}}}-{\widehat{{\varvec{y}}}}_{{\varvec{i}}{\varvec{l}}{\varvec{t}}}\right)-\stackrel{-}{\left({{\varvec{y}}}_{{\varvec{i}}{\varvec{l}}}-{\widehat{{\varvec{y}}}}_{{\varvec{i}}{\varvec{l}}}\right)}\right)*\left(\left({{\varvec{y}}}_{{\varvec{i}}{\varvec{l}},{\varvec{t}}-1}-{\widehat{{\varvec{y}}}}_{{\varvec{i}}{\varvec{l}}{\varvec{t}}}\right)-\stackrel{-}{\left({{\varvec{y}}}_{{\varvec{i}}{\varvec{l}}}-{\widehat{{\varvec{y}}}}_{{\varvec{i}}{\varvec{l}}}\right)}\right)\right)}{\frac{1}{n-1}*\sum_{t=1}^{n}{\left(\left({{\varvec{y}}}_{{\varvec{i}}{\varvec{l}}{\varvec{t}}}-{\widehat{{\varvec{y}}}}_{{\varvec{i}}{\varvec{l}}{\varvec{t}}}\right)-\stackrel{-}{\left({{\varvec{y}}}_{{\varvec{i}}{\varvec{l}}}-{\widehat{{\varvec{y}}}}_{{\varvec{i}}{\varvec{l}}}\right)}\right)}^{2}}$$

### Genetic analysis

The resilience indicators described above were genetically analyzed separately for each breed and two different lactation groups. The first one, labelled $P$ for primiparous, included phenotypes from the first lactation. For the second one, named $M$ for multiple lactations, we added information also from higher lactations of the cows in $P$ as repeated measurements. Thus, a univariate analysis was performed for each of six groups: first lactation HF, all lactation HF, first lactation FV, all lactation FV, first lactation BS, all lactation BS. The animal model for the data sets with information about cows in the first lactation was:

$$y=Xb+Zu+e$$

with $y$ vector of observations, $u$ vector of additive genetic effects, $e$ vector of residuals, and incidence matrices $X$ and $Z$. The animal model for all lactations of the considered cows was:

$$y=Xb+Zu+Wpe+e$$

where $pe$ is the vector of permanent environmental effects and $W$ is the incidence matrix. The additive genetic effects $u$, the permanent environmental effects $pe$ and the residual effects $e$ were normally distributed with $u \sim N(0,G{\sigma }_{u}^{2})$, $pe \sim N\left(0,{I}_{pe}{\sigma }_{pe}^{2}\right)$ and $e \sim N\left(0,{I}_{e}{\sigma }_{e}^{2}\right)$, whereby $I$ are identity matrices. The genomic relationship matrix $G$ was built according to VanRaden et al. [26]. The vector of fixed effects $b$ comprises in each model age at first calving in month, herd-year-season effect and completeness of lactation data. In the second model the effect of the lactation separated in first and higher lactation was added. Season in herd-year-season was divided into spring from March to May, summer from June to August, autumn from September to November, and winter from December to February and completeness of lactation data was graded in ≥ 90%, 80%—< 90%, 70%—< 80%, 60%—< 70%, 50%—< 60%. In all analyses, effect levels with fewer than six animals are excluded. This left 1,370 HF cows for $P$ and 1,538 HF cows for $M$, for FV 1,135 cows in $P$ and 1,551 in $M$ and for BS $P$ includes data from 310 and $M$ from 488 cows. For further analysis, we de-regressed the cow’s additive genetic effects, i.e. their GEBV, by dividing the effects by their reliabilities and standardized them to a mean of 0 and a standard deviation of 1 within each group. We also calculated the heritability as the proportion of additive genetic variance of the total variance.

Additionally, a selection index ($SI$) was computed that combines the resilience indicators into a single trait. The selection index is a linear combination of the GEBVs of individual resilience indicators, with weights chosen to optimally describe overall resilience. Weighting factors were determined in Keßler et al. [8], by maximizing the selection response in health traits when selecting on resilience indicators based on the performance trait daily milk yield. The weighting factors were adopted from this study, which were estimated in the same data set [8]. The selection index was calculated as

$$SI\left({\mathbf{y}}_{i}\right)=0.65*{\widetilde{v}}_{d}\left({\mathbf{y}}_{i}\right)+ 0.35* {\widetilde{v}}_{r}\left({\mathbf{y}}_{i}\right),$$

where ${\widetilde{v}}_{d}\left({\mathbf{y}}_{i}\right)$ and ${\widetilde{v}}_{r}\left({\mathbf{y}}_{i}\right)$ were GEBVs of the logarithmized, negated ${v}_{d}\left({\mathbf{y}}_{i}\right)$ and ${v}_{r}\left({\mathbf{y}}_{i}\right)$, for cow $i$. The selection index was calculated in both lactation groups, $P$ and $M$, and all three breeds.

### Imputation

For further analyses we imputed the 50 K genotypes to high-density genotypes. The following reference panels were used. For HF, we used data from 1,278 Australian Holsteins with 585,517 SNPs that were genotyped with the BovineHD Genotyping BeadChip [27]. High-density data from 3,439 FV cows and bulls including 629,028 SNPs genotyped with Illumina BovineHD Bead chip [28] and 192 BS individuals with 613,140 SNPs genotyped with Illumina Bovine-HD [23] were available. Similar to the 50 K genotype data, SNPs whose MAF was < 0.03, whose missing call rate was > 0.01 or which were found on sex chromosomes were removed. The positions of considered SNPs were updated to the reference genome ARS-UCD1.2 [24]. 522,726 SNPs in HF, 580,873 SNPs in FV and 503,971 SNPs in BS remained.

Reference panel data were phased using beagle 5.4 within each dataset. 50 K and high-density data of each breed were concorded with *conform-gt* and then imputed with beagle 5.4. According to [29], the effective population size Ne was set to 100. Apart from that, the default settings were used. SNPs with a dosage R-Squared < 0.75 are removed to maintain a sufficiently high imputation quality [19]. The final imputed data set contains 2,300 HF cows with 503,263 SNPs, 2,330 FV cows with 569,056 SNPs and 1,073 BS cows with 493,637 SNPs. For the across-breed population analysis, the imputed genotypes were merged with PLINK 1.9 [30], leaving 401,491 SNPs shared across all breeds.

### Population analysis

Population analyses were done in PLINK 1.9 for MDS and PLINK 2.0 for LD analysis [17]. Genomic relationship between the breeds was studied with a MDS based on imputed genotypes, considering only SNPs that remained in all three breeds after all filtering steps described above. The LD was analysed across breeds and breed-specific, using the maximum number of available SNPs in the imputed genotype datasets of each breed, respectively the merged imputed dataset after all filtering steps. The LD was calculated as the ${r}^{2}$ [31]:

$${r}^{2}= \frac{{\left({p}_{{A}_{1}{B}_{1}}-{p}_{{A}_{1}}{p}_{{B}_{1}}\right)}^{2}}{{p}_{{A}_{1}}{p}_{{A}_{2}}{p}_{{B}_{1}}{p}_{{B}_{2}}}$$

with $p$ the allele frequency, ${A}_{1}$ and ${A}_{2}$ alleles on gene locus $A$ and ${B}_{1}$ and ${B}_{2}$ alleles on gene locus $B$. The LD between pairs of SNPs that were a maximum of 1,000 SNPs or 3,000 kilobases apart was calculated.

### Genome-wide association study

For each of the six groups defined above, a GWAS was performed for the resilience indicator traits and the selection index. We optimized the GWAS model by minimizing the difference of the genomic inflation factor lambda ${\lambda }_{GC}$ to 1 in each breed. ${\lambda }_{GC}$ is calculated as the ratio between the median of the observed chi-squared test statistics and the expected median of 0.455 [32]. The value should be close to 1 or slightly above [22, 33]. We therefore decided for a mixed linear model based association analysis ($MLMA$) for HF, a $MLMA$ excluding the GRM of the chromosome where the SNP is located ($MLM-LOCO$) for BS and a $MLM-LOCO$ corrected for a fixed effect of the principal components calculated based on the whole genome (${\text{MLM}-\text{LOCO}}_{PC Genome}$) to account for population stratification for FV [21] (${\lambda }_{GC}$ of the selected GWAS are provided in the results, all ${\lambda }_{GC}$ are provided in Additional File 2).

GWAS were performed using the following model:

$$y = \mu + Xb+ Zu + e,$$

with $y$ vector of de-regressed, standardized additive genetic effects of each animal derived from univariate analysis for each resilience indicator trait and the selection index, the population mean $\upmu$, fixed effects $b$ with the incidence matrix $X$, including the reference allele coded with 0, 1 or 2, the vector of additive genetic effects $u$ with $u \sim N(0,G{\sigma }_{u}^{2})$ and $G$ the genomic relationship matrix created according to Yang et al. [34], and the residual term $e$ with $e \sim N(0,{{I}_{e}\sigma }_{e}^{2})$. In $MLM-LOCO$ and ${\text{MLM}-\text{LOCO}}_{PC Genome}$, $G$ is calculated for each chromosome separately and leaves out the chromosome, where the SNP to be tested for association is located. The vector $b$ includes the additive effect of the candidate SNP to be tested for association and for ${\text{MLM}-\text{LOCO}}_{PC Genome}$ in FV a covariate of 20 principal components, which were calculated based on the whole genome with software GCTA [35]. For the calculation of GWAS in GCTA, the reference allele was specified in accordance with ARS-UCD1.2.

For across-breed evaluation, we pooled the p-values in data set $P$ and data set $M$ per resilience indicator using the package *poolr* in R [36]. We used the Fisher method, which is based on the chi^2^-statistic:

$${X}^{2}=-2\sum_{i=1}^{k}\text{ln}({p}_{i})$$

with K number of tests (three) and pi the *p*-values of the GWAS.

The results of the single GWAS and pooled GWAS are shown as Manhattan plots, whereby two significance thresholds were defined. Genome-wide significance was determined using Bonferroni correction with, with $N$ number of SNPs, and a less stringent, suggestive level of significance was set to $p\le \frac{0.05}{N}$. For SNPs showing significant association at the stringent significance level, the Cattle QTL Database release 53 [27] was used to search for QTL in the range from 25 kB up- and downstream.

## Results

### Heritabilities

Heritabilities were in the range of 0.1 to 0.15 for ${v}_{d}$. BS showed the highest ${h}^{2}$ with 0.23 (SE = 0.15) in P and 0.15 (SE = 0.07) in M. HF and FV were similar with 0.13 (SE = 0.05) and 0.12 (SE = 0.06) in P and 0.1 (SE = 0.03) and 0.13 (SE = 0.03) in M. Repeatability was 0.26 (SE = 0.02) in HF, 0.25 (SE = 0.03) in FV and 0.19 (SE = 0.05) in BS. For ${v}_{r}$ FV showed the lowest h^2^ with 0.04 (SE = 0.04) in P and 0.08 (SE = 0.03) in M, and BS the highest h^2^ with 0.18 (SE = 0.15) in P and 0.2 (SE = 0.08) in M. HF ranged between them with 0.13 (SE = 0.04) in P and 0.15 (SE = 0.03) in M. Repeatabilities were higher than for ${v}_{d}$, with 0.32 (SE = 0.02) in HF, 0.35 (SE = 0.03) in FV and 0.4 (SE = 0.05) in BS. Estimates of ${h}^{2}$ for ${r}_{Auto}$ were rarely significant and low with values between 0.03 in FV and 0.12 in BS. Repeatability’s were 0.17 (SE = 0.02) for HF, 0.2 (SE = 0.03) for FV and 0.27 (SE = 0.06) for BS. Reliabilities for GEBVs were smaller in P data sets than in M data sets with an average difference of 0.1. The highest values in M were estimated in HF (0.37) and BS (0.33) for ${v}_{r}$ and the lowest in HF (0.15) and FV (0.14). Reliabilities of ${v}_{d}$ in M ranged around 0.29. Genetic correlation between the resilience indicator traits were analysed in a previous study and can be seen in Keßler et al. [6].

### Population analyses

**Fig. 1:**
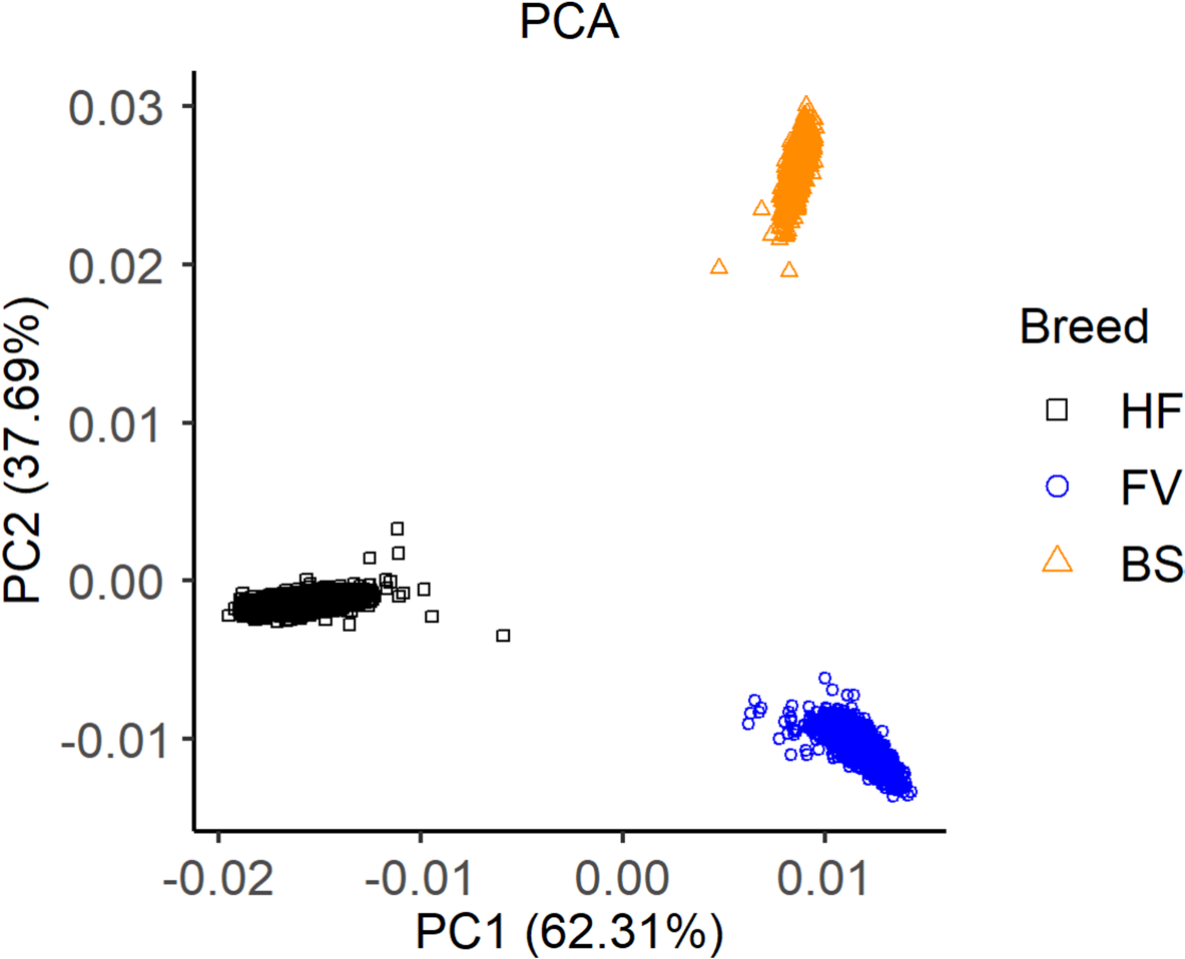
Genomic relationship between the breeds HF, FV and BS represented in a MDS-plot

The MDS (Fig. 1) shows three distinct clusters, each corresponding to one breed. The distances between the clusters are similar.

**Fig. 2:**
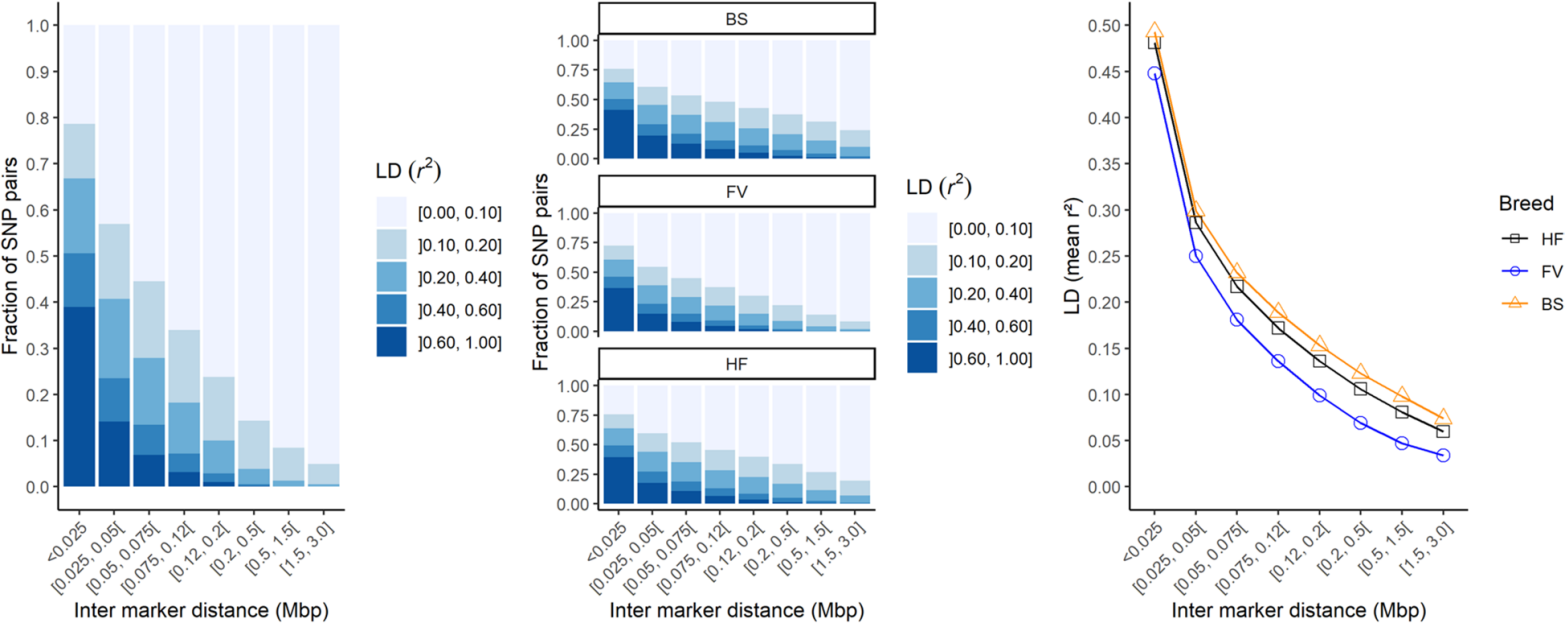
The decay of mean r.^2^ as a function of physical distance for breeds HF, FV and BS analyzed jointly (left), separated by breed (middle) and compared between the breeds (right)

The ${r}^{2}$ of the LD decreases with increasing marker distance (Fig. 2). This can be observed across all breeds as well as within breeds. The strongest decline in LD can be observed in FV, the lowest in BS. The LD within the breeds is similar, which means that the across-breed analysis showed comparable results.

### Genome-wide association study

Table 1 shows the values of ${\lambda }_{GC}$ for $MLMA$ in HF, $MLM-LOCO$ in BS and ${MLM-LOCO}_{PC Genome}$ in FV. Based on these values, we decided on the respective method for the GWAS. Our aim was to detect significant SNPs as accurately as possible without the inflation of false-positive results, therefore ${\lambda }_{GC}$ should be close to 1 or slightly above. Since the other methods considered led to a strong inflation of false-positive results (${\lambda }_{GC}$ >1.5) in HF [see Additional File 2], we decided to use the conservative MLMA approach.

**Table 1:** Genomic inflation factors ${\lambda }_{GC}$ for GWAS in German Holstein, German Fleckvieh, German Brown Swiss and pooled data sets of first lactation ($P$) and first and higher lactations ($M$) for resilience indicator traits

**German Holstein**		**German Fleckvieh**		**German Brown Swiss**		**Across-Breed**	
$P$	$M$	$P$	$M$	$P$	$M$	$P$	$M$
0.811	0.805	1.197	1.374	1.140	1.242	1.081	1.253
0.811	0.810	1.155	1.373	1.153	1.191	1.084	1.251
0.842	0.823	1.117	1.353	1.103	1.164	1.048	1.233
0.822	0.807	1.185	1.374	1.142	1.198	1.099	1.235

${v}_{d}$– variance of deviations between observed and predicted absolute daily milk yield

${v}_{r}$– variance of relative daily milk yield

${r}_{Auto}$– autocorrelation of deviations between observed and predicted absolute daily milk yield

$SI$– selection index resilience composed of single resilience indicators

**Fig. 3:**
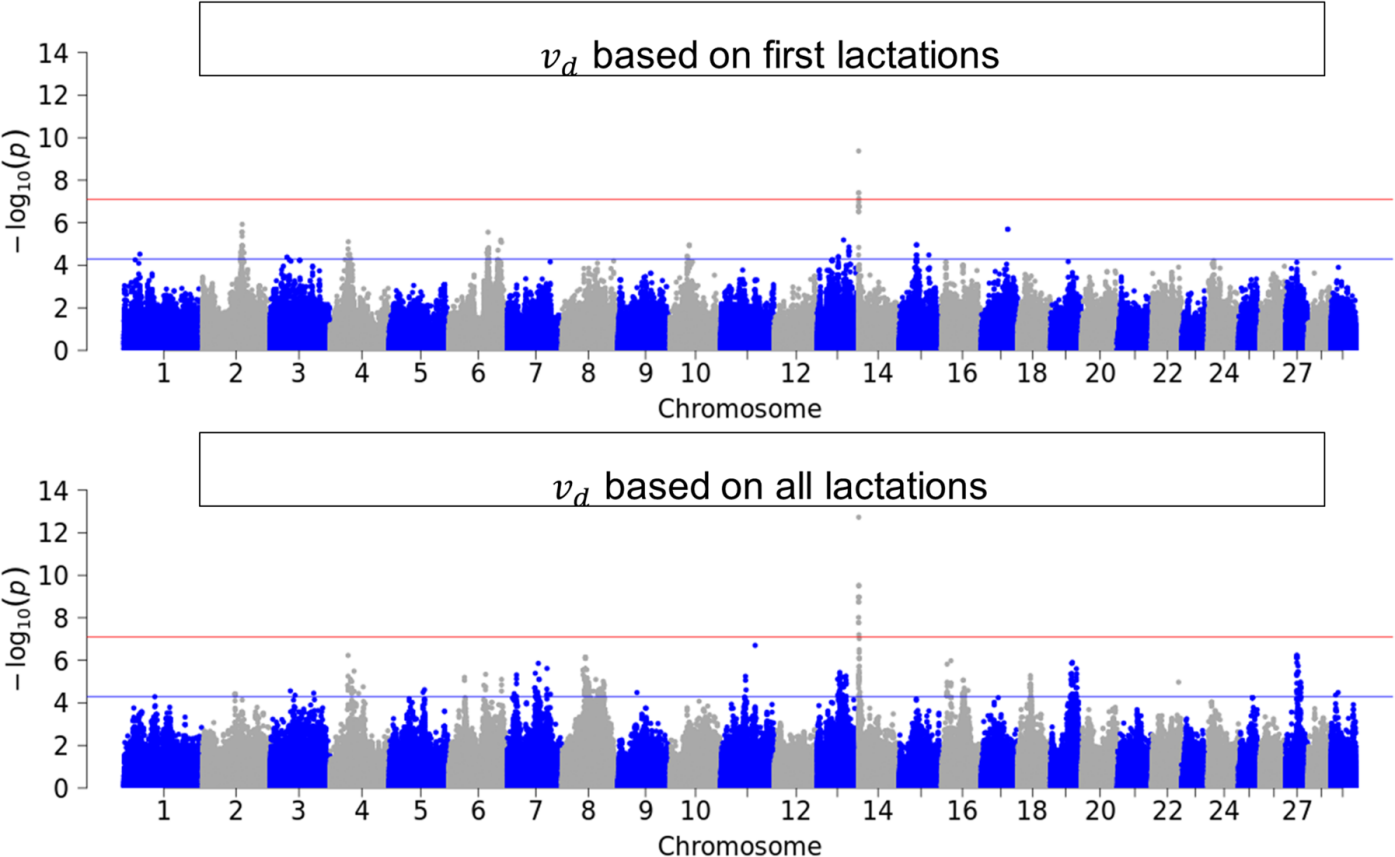
Manhattan-Plot of pooled p-values from single GWAS in the three breeds

**Fig. 4:**
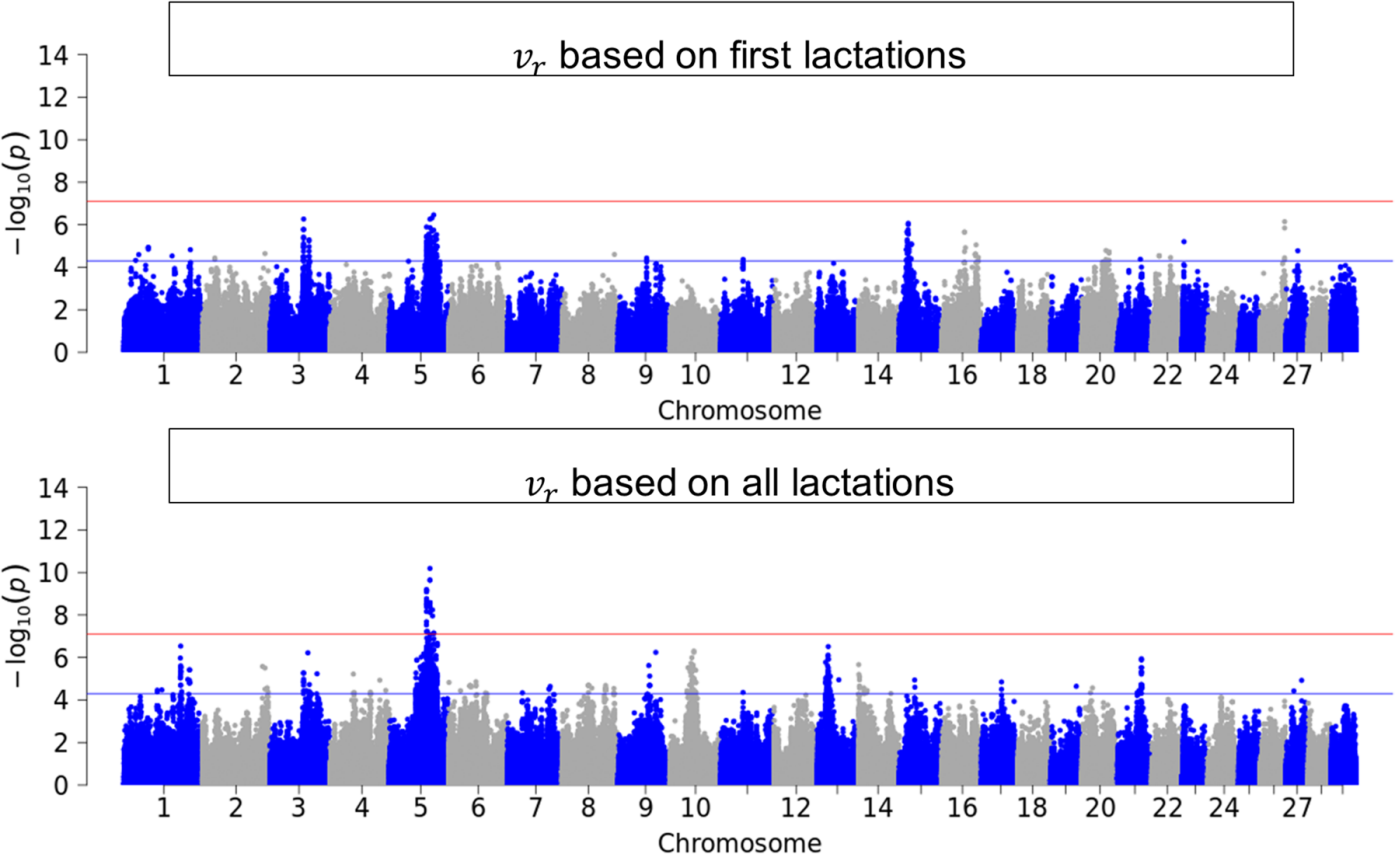
Manhattan-Plot of pooled p-values from single GWAS in the three breeds German Holstein, German Fleckvieh and German Brown Swiss for the resilience indicator trait ${v}_{r}$, which is the variance of relative daily milk yield in first lactation (top) and first and higher lactations (bottom)

**Fig. 5:**
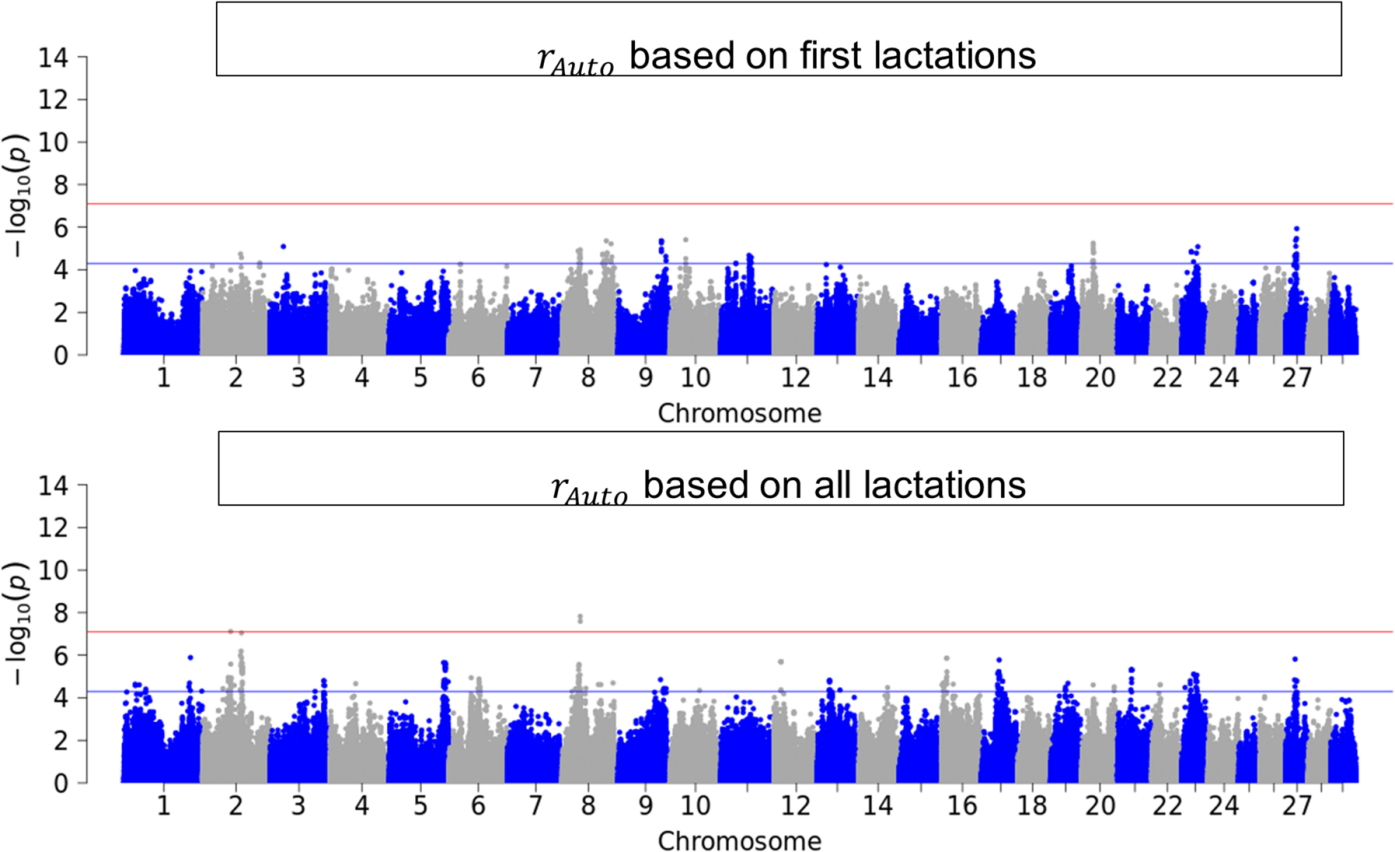
Manhattan-Plot of pooled p-values from single GWAS in the three breeds German Holstein, German Fleckvieh and German Brown Swiss for resilience indicator trait ${r}_{Auto}$, which is the autocorrelation in first lactation (top) and first and higher lactations (bottom)

**Fig. 6:**
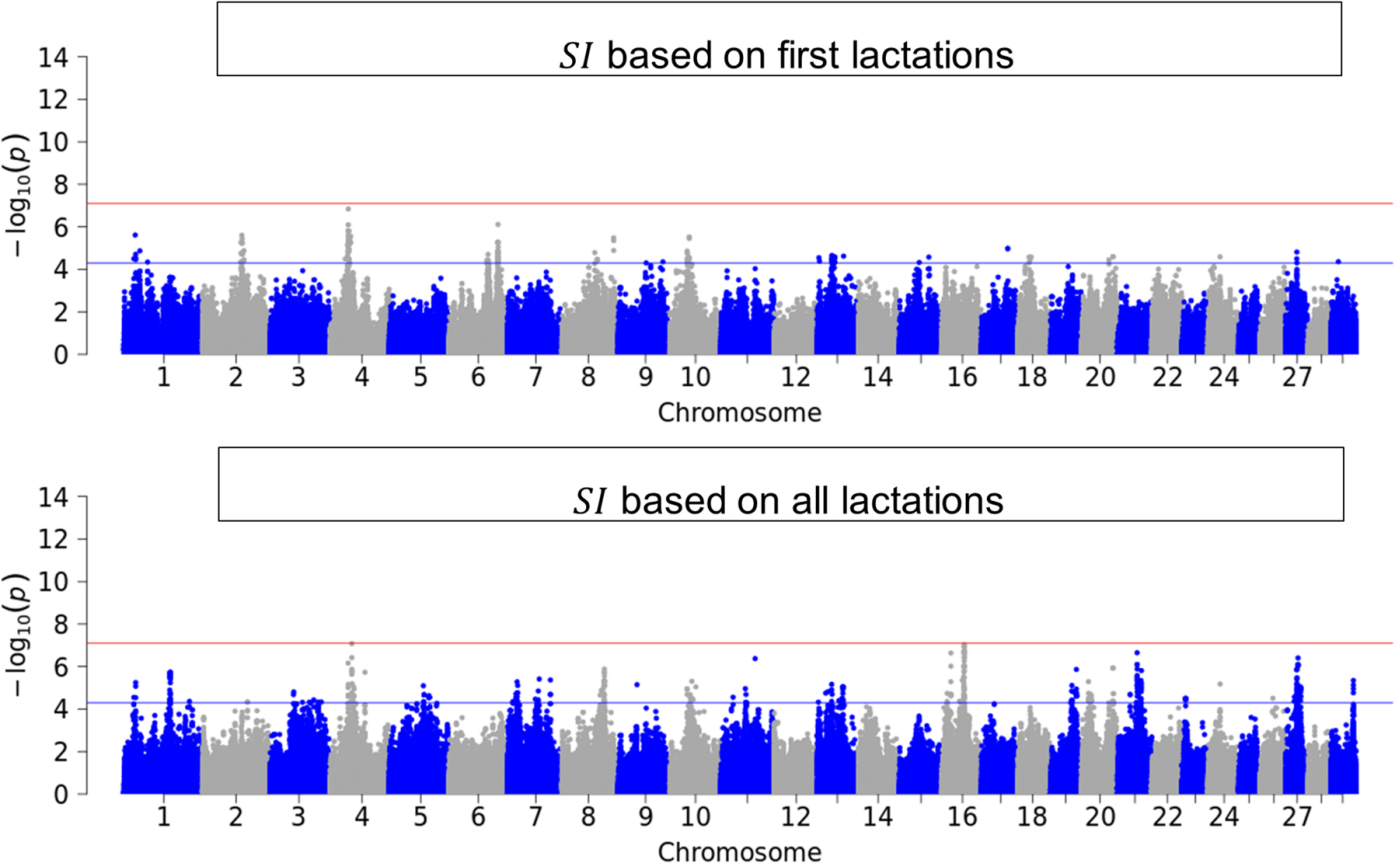
Manhattan-Plot of pooled p-values from single GWAS in the three breeds German Holstein, German Fleckvieh and German Brown Swiss for the resilience selection index $SI$ computed from resilience indicator traits calculated from daily milk yield in first lactation (top) and first and higher lactations (bottom)

Figures 3, 4, 5 and 6 illustrate the results of GWAS for each resilience indicator trait after pooling p-values across breeds for first and for all available lactations. Genome-wide significant SNPs were found for ${v}_{d}$ in the first lactation and for all single resilience indicator traits in the data set with all available lactations. SNPs that reached the suggestive significance threshold were found for all traits and groups, also for $SI$. These SNPs were distributed throughout the genome, with the variance-based indicators showing more significant SNPs. For the sake of completeness, the Manhattan plots can be viewed separately by breed in the Supplementary (see Additional File 4).

Number of suggestive and genome-wide significant SNPs can be seen in Table 2, while the name of the genome-wide significant SNPs and their base pair positions can be viewed in the Supplementary [see Additional File 3]. According to previous studies [e.g. [37, 38], most correlated QTL potentially influence milk yield, reproductive performance, body condition or longevity. Furthermore, QTL associated with udder, metabolic and claw health as well as immunological parameters were found (see Additional File 3 for an overview).

**Table 2:** Number of significant SNPs for resilience indicator traits based on suggestive ($p < 5*{10}^{-5}$) and genomewide ($p \le \frac{0.05}{Number of SNPs}$) significance threshold for first lactation of ($P$) and for first and higher lactations of cows ($M$)

**Significance of the SNP**	${{\varvec{v}}}_{{\varvec{d}}}$		${{\varvec{v}}}_{{\varvec{r}}}$		${{\varvec{r}}}_{{\varvec{A}}{\varvec{u}}{\varvec{t}}{\varvec{o}}}$		${\varvec{S}}{\varvec{I}}$	
	${\varvec{P}}$	${\varvec{M}}$	${\varvec{P}}$	${\varvec{M}}$	${\varvec{P}}$	${\varvec{M}}$	${\varvec{P}}$	${\varvec{M}}$
Genome-wide	4	13	0	38	0	3	0	0
Suggestive	93	379	214	716	84	234	196	441

${v}_{d}$ – variance of deviations between observed and predicted absolute daily milk yield

${v}_{r}$ – variance of relative daily milk yield

${r}_{Auto}$ – autocorrelation of deviations between observed and predicted absolute daily milk yield

$SI$ – selection index resilience composed of single resilience indicators

## Discussion

### General discussion

In Germany, there are hardly any studies that compare and, above all, combine the genomic data from HF, FV and BS, likely also due to the division of breeding value estimation between different organisations. We matched the three data sets, which enabled us to increase our data basis and thus our power. In addition, we can draw conclusions about all three breeds, whereas analysing the BS alone would have been critical due to the small number of individuals. Multibreed analyses lead to a higher accuracy of estimates for breeds with small reference populations [39], like BS, and also allow estimates for crossbred animals, for example when estimating genomic breeding values [40]. For purebred, large reference populations a single breed analysis would be more advantageous [41], which is why we did a two-step analysis: run the mixed-linear model and the genome-wide association study for each breed in the first step, and combine the results in a second step.

Heritabilities in our data set were in the range with previous studies with Holstein Friesian [4, 5, 10]. Higher heritabilities with higher standard errors in BS could be due to the limited number of individuals and farms, which is why fewer estimations of variance components per trait were significant. The inclusion of all lactations of the individuals compared to only the first lactation tended to explain a higher proportion of the total variance, captured by the permanent environment effect.

### Population analysis

The breeds studied are genetically different, which was as expected and previously reported for the same breeds in Switzerland [42]. The number of SNPs considered was > 300,000 and the decrease in LD with increasing distance between SNPs was similar in all breeds. Thus, all three breeds could be analysed and discussed jointly in the GWAS [43]. For the subsequent search for QTL associated with the genome-wide significant SNPs for resilience indicators, a window had to be defined. This is limited by the useful LD between SNP and QTL, which was described by de Roos et al. [40] and Sargolzaei et al. [44] with ${r}^{2}$ >0.3. In our study, we observed a mean ${r}^{2}$>0.4 in a distance of up to 25 kb. In addition, as this was an across-breed study, we were more stringent with the window size.

### Genome-wide association study

The inflation factor ${\lambda }_{GC}$ was close to 1 or slightly above for most of the data sets (Table 1) and only for FV first and higher lactations above 1.3. This suggests that the p-values of the GWAS might be slightly inflated for the latter, but not for the remaining data sets. A conservative approach with ${\lambda }_{GC}$ <1 was chosen for HF, as the other methods tested resulted in highly inflated results. The pooled, across-breed analysis showed that resilience indicators are polygenic traits influenced by many gene loci. This was shown by the high number of suggestive significant SNPs distributed across the entire genome and the small number of genome-wide significant SNPs. Similar results were reported by previous studies about resilience indicators in laying hens [15], pigs [14] and dairy cows [13]. Chen et al. [13] reported a strong peak on *BTA14*, whereby the milk yield level had a significant causal effect on the performance variability over several lactations. QTL that are closely adjacent to our genome-wide significant SNPs simultaneously influence several traits of performance, fertility and health [45]. The inclusion of repeated measurements per individual led to a higher power of the GWAS. In contrast to the data set of the first lactation, genome-wide significant SNPs were found for ${r}_{Auto}$ and ${v}_{r}$ in the data set including all lactations. For ${v}_{d}$, there was an increase in the number of genome-wide significant SNPs when all lactations were considered rather than just the first. However, they did not share their genetic backgrounds, i.e. different genome regions influence different resilience indicators. This was previously described in Chen et al. [13] for traits similar to our ${v}_{d}$ and ${r}_{Auto}$ and were expected from us, because previous analysis of genetic correlation between single resilience indicator traits showed only less to moderate correlation, which can be interpreted as different traits[6].

### Common genetic background of resilience and other traits

Genomic regions that have a strong effect on our resilience indicators influence milk yield, milk ingredients, health and fertility traits. A significant genetic effect on the resilience indicator ${v}_{r}$ occurs from *BTA5*, in the region of the genes *CACNG2*, *EIF3D*, *IFT27* and *PVALB*, which influence milk yield, and next to *5s_RNA*, which influences the amount of milk fat [37]. With *7SK*, ${r}_{Auto}$ on *BTA8* also has a milk yield-regulating gene in the analysed window. A significant effect of ${v}_{d}$ were detected on *BTA14* in a region in which pleiotropic genes have an effect on milk quantity and ingredients, especially milk fat acids [46]. Known genes are for example *DGAT1*, *ARHGAP39*, *bta-mir-2308*, *LRRC24*, *CPSF1*, *RECQL4*, *PLEC*, *PPP1R16A*, *MFSD3*, *GPT*, *KIFC2*, *CYHR1*, *TONSL* or *FOXH1* [37, 38, 45]. A strong antagonism between the SNP effects on the amount of milk fat and the amount of milk and milk protein yield is known for *PLEC* and *PPP1R16A* [38]. An unbalanced fat-to-protein ratio can be a sign of a negative energy balance and metabolic diseases [47, 48], which is why animals with a strong genetic antagonism may be less resilient. In addition, this shared genetic background of performance and resilience influences the stability of milk and milk fat yield under heat stress [49] and the overall profitability of a dairy cow [50], which was also described in Poppe et al. [51].

Jiang et al. [38] reported a QTL next to *MRPS35* on *BTA5*, which is in a high LD with genome-wide significant SNPs for ${v}_{r}$, influencing pregnancy and conception rate, and Cochran et al. [45] a QTL on *CPSF1*, influencing conception rate and ${v}_{d}$. Galliou et al. [52] detected on *BTA2* in a genomic region with an effect on ${r}_{Auto}$, QTL for heifer reproduction traits that may also affect reproduction in cows. Health indicator traits like the somatic cell score for mastitis [45] or the concentration of beta-hydroxybutyrat (BHB) in milk for ketosis [53] are affected by genomic regions on *BTA14*, also influencing ${v}_{d}$. This is confirmed by a study with the EBV of mastitis, showing a significant influence in the same genomic area [54].

The selection index $SI$ was not influenced by genome-wide significant SNPs, but strong suggestive peaks emerged on *BTA4*, *BTA16*, *BTA21* and *BTA27.* QTL on these chromosomes influence milk yield and especially milk fat, longevity and reproduction ability. This underlines the polygenic characteristics of resilience.

### Breeding for resilience

Breeding for resilience to increase recovery speed and reduce performance variability reduces sensitivity to external disturbances. The current approach of using indicator traits calculated on the basis of longitudinal data seems promising: moderate heritabilities and desirable correlations with other traits have been shown in previous studies [4–7, 10, 55]. The combination of different indicator traits is suitable for integration into practical breeding [11]. For example, the weighting factors can be determined by maximizing selection response in functional and health traits. In this way, performance and functionality are combined and mediated. Because of favorable genetic correlations, breeders could use this EBV in addition to the total merit index to more easily identify sires that combine yield, longevity and health [8].

There are two main points in favor of breeding for resilience: First, no extra data collection is necessary for breeding value estimation [2]. Second, in some breeds, little or no health traits are recorded; breeding for resilience could improve animal health [6, 8]. A homogeneous, healthy herd with stable performance is easier to manage and more profitable [2, 51]. In the future, the suitability of other statistical parameters as resilience indicator traits [12] and new longitudinal measured phenotypes (e.g. activity data, omics data) will be studied [7, 56].

## Conclusion

Our study demonstrated clear genetic differentiation between German populations of HF, FV, and BS breeds, while showing similar patterns of LD decay across breeds. Significant heritabilities for resilience indicators were estimated across all three breeds. GWAS results revealed the polygenic nature of resilience, with significant SNPs distributed across multiple BTA. Genome-wide significant SNPs for ${v}_{d}$ were found on *BTA14*, for ${v}_{r}$ on *BTA5* and for ${r}_{Auto}$ on *BTA2* and BTA8, which indicate a different genetic background. A selection index combined of ${v}_{d}$ and ${v}_{r}$ was mainly influenced by SNPs on *BTA4*, BTA*16*, BTA*21* and BTA*27.* We found strong links with QTL affecting production, health and fertility, which confirms the complex genetic architecture of resilience. Notably, analyzing resilience across multiple lactations increased statistical power and revealed additional significant associations compared to first-lactation analysis.

## Supplementary Information

Below is the link to the electronic supplementary material.

Supplementary Material 1.

Supplementary Material 2.

Supplementary Material 3.

Supplementary Material 4.

## Data Availability

The data that support the findings of this study are available upon request and after signing a material transfer agreement with the cattle breeding organization Rinderunion Baden-Württemberg e.V. (Herbertingen, Germany). GWAS summary statistics can be shared without any restrictions. For data requests, please contact JB.

## Abbreviations

BS: German Brown Swiss
FV: German Fleckvieh
HF: German Holstein
P: First lactation
M: First and higher lactation
${r}_{Auto}$: Resilience indicator trait (autocorrelation of deviations between observed and predicted absolute daily milk yield)
$SI$: Selection index resilience composed of single resilience indicators
${v}_{d}$: Resilience indicator trait (variance of deviations between observed and predicted absolute daily milk yield)
${v}_{r}$: Resilience indicator trait (variance of relative daily milk yield)

## References

[1] Hansen J, Sato M, Ruedy R. Perception of climate change. Proc Natl Acad Sci U S A. 2012;109:E2415–23. 10.1073/pnas.1205276109. 22869707 10.1073/pnas.1205276109 PMC3443154

[2] Berghof TVL, Poppe M, Mulder HA. Opportunities to improve resilience in animal breeding programs. Front Genet. 2018;9:692. 10.3389/fgene.2018.00692. 30693014 10.3389/fgene.2018.00692 PMC6339870

[3] Colditz IG, Hine BC. Resilience in farm animals: biology, management, breeding and implications for animal welfare. Anim Prod Sci. 2016;56:1961–83. 10.1071/AN15297.

[4] Chen S-Y, Boerman JP, Gloria LS, Pedrosa VB, Doucette J, Brito LF. Genomic-based genetic parameters for resilience across lactations in North American Holstein cattle based on variability in daily milk yield records. J Dairy Sci. 2023;106:4133–46. 10.3168/jds.2022-22754. 37105879 10.3168/jds.2022-22754

[5] Poppe M, Veerkamp RF, van Pelt ML, Mulder HA. Exploration of variance, autocorrelation, and skewness of deviations from lactation curves as resilience indicators for breeding. J Dairy Sci. 2020;103:1667–84. 10.3168/jds.2019-17290. 31759590 10.3168/jds.2019-17290

[6] Keßler F, Wellmann R, Chagunda MGG, Bennewitz J. Resilience indicator traits in three dairy cattle breeds in Baden-Württemberg. J Dairy Sci. 2024. 10.3168/jds.2023-24305. 38310955 10.3168/jds.2023-24305

[7] van Dixhoorn I, de Mol RM, Schnabel SK, van der Werf J, van Mourik S, Bolhuis JE, et al. Behavioral patterns as indicators of resilience after parturition in dairy cows. J Dairy Sci. 2023. 10.3168/jds.2022-22891. 37500445 10.3168/jds.2022-22891

[8] Keßler F, Wellmann R, Chagunda MGG, Bennewitz J. Toward a resilience selection index with indicator traits in German Holstein dairy cattle. J Dairy Sci. 2025;108:726–34. 10.3168/jds.2024-25323. 39694257 10.3168/jds.2024-25323

[9] Wang A, Brito LF, Zhang H, Shi R, Zhu L, Liu D, et al. Exploring milk loss and variability during environmental perturbations across lactation stages as resilience indicators in Holstein cattle. Front Genet. 2022;13:1031557. 10.3389/fgene.2022.1031557. 36531242 10.3389/fgene.2022.1031557 PMC9757536

[10] Wang A, Su G, Brito LF, Zhang H, Shi R, Liu D, et al. Investigating the relationship between fluctuations in daily milk yield as resilience indicators and health traits in Holstein cattle. J Dairy Sci. 2024;107:1535–48. 10.3168/jds.2023-23495. 37690717 10.3168/jds.2023-23495

[11] Meijer N, Bouwmeester-Vosman J, van Pelt M, de Jong G. Breeding for resilience in the Netherlands and Flanders: INTERBULL; 2024.

[12] Ghaderi Zefreh M, Pong-Wong R, Doeschl-Wilson A. Validating statistical properties of resilience indicators derived from simulated longitudinal performance measures of farmed animals. Animal. 2024;18:101248. 10.1016/j.animal.2024.101248. 39096601 10.1016/j.animal.2024.101248

[13] Chen SY, Gloria LS, Pedrosa VB, Doucette J, Boerman JP, Brito LF. Unravelling the genomic background of resilience based on variability in milk yield and milk production levels in North American Holstein cattle through GWAS and Mendelian randomization analyses. J Dairy Sci. 2023. 10.3168/jds.2023-23650. 37776995 10.3168/jds.2023-23650

[14] Mancin E, Maltecca C, Jiang J, Huang YJ, Tiezzi F. Capturing resilience from phenotypic deviations: a case study using feed consumption and whole genome data in pigs. BMC Genomics. 2024;25:1128. 10.1186/s12864-024-11052-0. 39574040 10.1186/s12864-024-11052-0 PMC11583387

[15] Doekes HP, Bovenhuis H, Berghof TVL, Peeters K, Visscher J, Mulder HA. Research note: Genome-wide association study for natural antibodies and resilience in a purebred layer chicken line. Poult Sci. 2023;102(1):102312. 10.1016/j.psj.2022.102312. 36473374 10.1016/j.psj.2022.102312 PMC9720488

[16] R Core Team. R: A language and environment for statistical computing. 2022. https://www.R-project.org/. Accessed 9 Oct 2023.

[17] Chang CC, Chow CC, Tellier LC, Vattikuti S, Purcell SM, Lee JJ. Second-generation PLINK: rising to the challenge of larger and richer datasets. Gigascience. 2015;4:7. 10.1186/s13742-015-0047-8. 25722852 10.1186/s13742-015-0047-8 PMC4342193

[18] Browning BL, Tian X, Zhou Y, Browning SR. Fast two-stage phasing of large-scale sequence data. Am J Hum Genet. 2021;108:1880–90. 10.1016/j.ajhg.2021.08.005. 34478634 10.1016/j.ajhg.2021.08.005 PMC8551421

[19] Browning BL, Zhou Y, Browning SR. A one-penny imputed genome from next-generation reference panels. Am J Hum Genet. 2018;103:338–48. 10.1016/j.ajhg.2018.07.015. 30100085 10.1016/j.ajhg.2018.07.015 PMC6128308

[20] Butler DG. Package ‘asreml’. 2018. https://asreml.kb.vsni.co.uk/wp-content/uploads/sites/3/2018/07/ASReml-Package.pdf.

[21] Yang J, Zaitlen NA, Goddard ME, Visscher PM, Price AL. Advantages and pitfalls in the application of mixed-model association methods. Nat Genet. 2014;46:100–6. 10.1038/ng.2876. 24473328 10.1038/ng.2876 PMC3989144

[22] Schneider H, Segelke D, Tetens J, Thaller G, Bennewitz J. A genomic assessment of the correlation between milk production traits and claw and udder health traits in Holstein dairy cattle. J Dairy Sci. 2023;106:1190–205. 10.3168/jds.2022-22312. 36460501 10.3168/jds.2022-22312

[23] Dreher C, Wellmann R, Stratz P, Schmid M, Preuß S, Hamann H, Bennewitz J. Genomic analysis of perinatal sucking reflex in German Brown Swiss calves. J Dairy Sci. 2019;102:6296–305. 10.3168/jds.2019-16487. 31056319 10.3168/jds.2019-16487

[24] Rosen BD, Bickhart DM, Schnabel RD, Koren S, Elsik CG, Tseng E, et al. De novo assembly of the cattle reference genome with single-molecule sequencing. Gigascience. 2020. 10.1093/gigascience/giaa021. 32191811 10.1093/gigascience/giaa021 PMC7081964

[25] Ramsey J, Ripley B. pspline: Penalized Smoothing Splines. 2022. https://cran.r-project.org/web/packages/pspline/index.html. Accessed 29 Nov 2023.

[26] VanRaden PM. Efficient methods to compute genomic predictions. J Dairy Sci. 2008;91:4414–23. 10.3168/jds.2007-0980. 18946147 10.3168/jds.2007-0980

[27] van den Berg I, Ho PN, Nguyen TV, Haile-Mariam M, MacLeod IM, Beatson PR, et al. GWAS and genomic prediction of milk urea nitrogen in Australian and New Zealand dairy cattle. Genet Sel Evol. 2022;54:15. 10.1186/s12711-022-00707-9. 35183113 10.1186/s12711-022-00707-9 PMC8858489

[28] Ertl J, Legarra A, Vitezica ZG, Varona L, Edel C, Emmerling R, Götz K-U. Genomic analysis of dominance effects on milk production and conformation traits in Fleckvieh cattle. Genet Sel Evol. 2014;46:40. 10.1186/1297-9686-46-40. 24962065 10.1186/1297-9686-46-40 PMC4230028

[29] Qanbari S, Pimentel ECG, Tetens J, Thaller G, Lichtner P, Sharifi AR, Simianer H. The pattern of linkage disequilibrium in German Holstein cattle. Anim Genet. 2010;41:346–56. 10.1111/j.1365-2052.2009.02011.x. 20055813 10.1111/j.1365-2052.2009.02011.x

[30] Purcell S, Neale B, Todd-Brown K, Thomas L, Ferreira MAR, Bender D, et al. PLINK: a tool set for whole-genome association and population-based linkage analyses. Am J Hum Genet. 2007;81:559–75. 10.1086/519795. 17701901 10.1086/519795 PMC1950838

[31] Hill WG, Robertson A. Linkage disequilibrium in finite populations. Theor Appl Genet. 1968;38:226–31. 10.1007/BF01245622. 24442307 10.1007/BF01245622

[32] van den Berg S, Vandenplas J, van Eeuwijk FA, Lopes MS, Veerkamp RF. Significance testing and genomic inflation factor using high-density genotypes or whole-genome sequence data. J Anim Breed Genet. 2019;136:418–29. 10.1111/jbg.12419. 31215703 10.1111/jbg.12419 PMC6900143

[33] Yang J, Weedon MN, Purcell S, Lettre G, Estrada K, Willer CJ, et al. Genomic inflation factors under polygenic inheritance. Eur J Hum Genet. 2011;19:807–12. 10.1038/ejhg.2011.39. 21407268 10.1038/ejhg.2011.39 PMC3137506

[34] Yang J, Benyamin B, McEvoy BP, Gordon S, Henders AK, Nyholt DR, et al. Common SNPs explain a large proportion of the heritability for human height. Nat Genet. 2010;42:565–9. 10.1038/ng.608. 20562875 10.1038/ng.608 PMC3232052

[35] Yin T, König S. Genome-wide associations and detection of potential candidate genes for direct genetic and maternal genetic effects influencing dairy cattle body weight at different ages. Genet Sel Evol. 2019;51:4. 10.1186/s12711-018-0444-4. 30727969 10.1186/s12711-018-0444-4 PMC6366057

[36] Cinar O, Viechtbauer W. The poolr package for combining independent and dependent *p* values. J Stat Soft. 2022;101:1–42. 10.18637/jss.v101.i01.

[37] Pedrosa VB, Schenkel FS, Chen S-Y, Oliveira HR, Casey TM, Melka MG, Brito LF. Genomewide association analyses of lactation persistency and milk production traits in Holstein cattle based on imputed whole-genome sequence data. Genes. 2021;12:1830. 10.3390/genes12111830. 34828436 10.3390/genes12111830 PMC8624223

[38] Jiang J, Ma L, Prakapenka D, VanRaden PM, Cole JB, Da Y. A large-scale genome-wide association study in U.S. Holstein cattle. Front Genet. 2019;10:412. 10.3389/fgene.2019.00412. 31139206 10.3389/fgene.2019.00412 PMC6527781

[39] Frischknecht M, Pausch H, Bapst B, Signer-Hasler H, Flury C, Garrick D, et al. Highly accurate sequence imputation enables precise QTL mapping in Brown Swiss cattle. BMC Genomics. 2017;18:999. 10.1186/s12864-017-4390-2. 29284405 10.1186/s12864-017-4390-2 PMC5747239

[40] Karaman E, Su G, Croue I, Lund MS. Genomic prediction using a reference population of multiple pure breeds and admixed individuals. Genet Sel Evol. 2021;53:46. 10.1186/s12711-021-00637-y. 34058971 10.1186/s12711-021-00637-y PMC8168010

[41] Misztal I, Steyn Y, Lourenco DAL. Genomic evaluation with multibreed and crossbred data. JDS Communications. 2022;3:156–9. 10.3168/jdsc.2021-0177. 36339739 10.3168/jdsc.2021-0177 PMC9623721

[42] Signer-Hasler H, Burren A, Neuditschko M, Frischknecht M, Garrick D, Stricker C, et al. Population structure and genomic inbreeding in nine Swiss dairy cattle populations. Genet Sel Evol. 2017;49:83. 10.1186/s12711-017-0358-6. 29115934 10.1186/s12711-017-0358-6 PMC5674839

[43] de Roos APW, Hayes BJ, Spelman RJ, Goddard ME. Linkage disequilibrium and persistence of phase in Holstein-Friesian, Jersey and Angus cattle. Genetics. 2008;179:1503–12. 10.1534/genetics.107.084301. 18622038 10.1534/genetics.107.084301 PMC2475750

[44] Sargolzaei M, Schenkel FS, Jansen GB, Schaeffer LR. Extent of linkage disequilibrium in Holstein cattle in North America. J Dairy Sci. 2008;91:2106–17. 10.3168/jds.2007-0553. 18420642 10.3168/jds.2007-0553

[45] Cochran SD, Cole JB, Null DJ, Hansen PJ. Discovery of single nucleotide polymorphisms in candidate genes associated with fertility and production traits in Holstein cattle. BMC Genet. 2013;14:49. 10.1186/1471-2156-14-49. 23759029 10.1186/1471-2156-14-49 PMC3686577

[46] Gebreyesus G, Buitenhuis AJ, Poulsen NA, Visker MHPW, Zhang Q, van Valenberg HJF, et al. Combining multi-population datasets for joint genome-wide association and meta-analyses: the case of bovine milk fat composition traits. J Dairy Sci. 2019;102:11124–41. 10.3168/jds.2019-16676. 31563305 10.3168/jds.2019-16676

[47] Toni F, Vincenti L, Grigoletto L, Ricci A, Schukken YH. Early lactation ratio of fat and protein percentage in milk is associated with health, milk production, and survival. J Dairy Sci. 2011;94:1772–83. 10.3168/jds.2010-3389. 21426966 10.3168/jds.2010-3389

[48] Buttchereit N, Stamer E, Junge W, Thaller G. Short communication: genetic relationships among daily energy balance, feed intake, body condition score, and fat to protein ratio of milk in dairy cows. J Dairy Sci. 2011;94:1586–91. 10.3168/jds.2010-3396. 21338824 10.3168/jds.2010-3396

[49] Macciotta NPP, Biffani S, Bernabucci U, Lacetera N, Vitali A, Ajmone-Marsan P, Nardone A. Derivation and genome-wide association study of a principal component-based measure of heat tolerance in dairy cattle. J Dairy Sci. 2017;100:4683–97. 10.3168/jds.2016-12249. 28365122 10.3168/jds.2016-12249

[50] Nayeri S, Sargolzaei M, Abo-Ismail MK, Miller S, Schenkel F, Moore SS, Stothard P. Genome-wide association study for lactation persistency, female fertility, longevity, and lifetime profit index traits in Holstein dairy cattle. J Dairy Sci. 2017;100:1246–58. 10.3168/jds.2016-11770. 27889128 10.3168/jds.2016-11770

[51] Poppe M, Veerkamp RF, Mulder HA, Hogeveen H. Observational study on associations between resilience indicators based on daily milk yield in first lactation and lifetime profitability. J Dairy Sci. 2022;105:8158–76. 10.3168/jds.2021-21532. 36028351 10.3168/jds.2021-21532

[52] Galliou JM, Kiser JN, Oliver KF, Seabury CM, Moraes JGN, Burns GW, et al. Identification of loci and pathways associated with heifer conception rate in U.S. Holsteins. Genes. 2020. 10.3390/genes11070767. 32650431 10.3390/genes11070767 PMC7397161

[53] Nayeri S, Schenkel F, Fleming A, Kroezen V, Sargolzaei M, Baes C, et al. Genome-wide association analysis for β-hydroxybutyrate concentration in milk in Holstein dairy cattle. BMC Genet. 2019;20:58. 10.1186/s12863-019-0761-9. 31311492 10.1186/s12863-019-0761-9 PMC6636026

[54] Sahana G, Guldbrandtsen B, Thomsen B, Lund MS. Confirmation and fine-mapping of clinical mastitis and somatic cell score QTL in Nordic Holstein cattle. Anim Genet. 2013;44:620–6. 10.1111/age.12053. 23647142 10.1111/age.12053

[55] Oloo R, Mrode R, Bennewitz J, Ekine-Dzivenu CC, Ojango JM, Gebreyohanes G, et al. Potential for quantifying general resilience of dairy cattle in sub-Saharan Africa using deviations in milk yield. Front Genet. 2023. 10.3389/fgene.2023.1208158. 38162680 10.3389/fgene.2023.1208158 PMC10757848

[56] Poppe M, Mulder HA, van Pelt ML, Mullaart E, Hogeveen H, Veerkamp RF. Development of resilience indicator traits based on daily step count data for dairy cattle breeding. Genet Sel Evol. 2022;54:21. 10.1186/s12711-022-00713-x. 35287581 10.1186/s12711-022-00713-x PMC8919560

