# Supplementary material for: Mapping genes for resilient dairy cows by means of across-breed genome-wide association analysis
Source: BMC Genomics. 2025 Oct 1;26:879. doi: 10.1186/s12864-025-11940-z (PMC12486977; doi:10.1186/s12864-025-11940-z)
Supplement: Supplementary file 1 — Supplementary Material 1. [file 12864_2025_11940_MOESM1_ESM.docx]

**Provision of data set sizes before and after filtering**

**Table 3:** Overview of the size of the phenotype and genotype data sets before and after processing

|  |  | BS | FV | HF |
| --- | --- | --- | --- | --- |
| Dataset phenotypes | Initial number of individuals | 3,608 | 8,943 | 8,474 |
|  | Initial number of lactations | 5,038 | 15,472 | 13,517 |
|  | Number of individuals after filtering for at least 50 % of data points per lactation | 1,465 | 4,614 | 4,204 |
|  | Number of lactations after filtering for at least 50 % of data points per lactation | 2,505 | 9,494 | 8,111 |
| Data set of genotypes | Initial number of genotypes 50K | 1,073 | 2,330 | 2,300 |
|  | Initial number of usable SNPs 50K | 38,482 | 40,864 | 45,613 |
|  | Number of individuals in the HD-genotyped reference sample | 192 | 3,439 | 1,278 |
|  | Initial number of useable SNPs HD | 613,140 | 629,028 | 585,517 |
|  | Number of imputed SNPs after excluding SNPs with MAF <0.03 | 493,637 | 569,056 | 503,263 |
| Univariate analysis | Number of individuals in analysis of first lactation (P) | 310 | 1,135 | 1,370 |
|  | Number of individuals in analysis of first and higher lactation (M) | 477 | 1,551 | 1,538 |
|  | Number of lactations in analysis of first and higher lactation (M) | 885 | 2,874 | 3,409 |
|  | Number SNPs considered in the relationship matrix | 31,527 | 34,238 | 31,416 |
| Genome-wide association analysis | Number of individuals in analysis of first lactation (P) | 310 | 1,135 | 1,370 |
|  | Number of individuals in analysis of first and higher lactation (M) | 477 | 1,551 | 1,538 |
|  | Number of lactations in analysis of first and higher lactation (M) | 885 | 2,874 | 3,409 |
|  | Number SNPs considered in the genome-wide association analysis | 493,637 | 569,056 | 503,263 |
