# Supplementary material for: Mapping genes for resilient dairy cows by means of across-breed genome-wide association analysis
Source: BMC Genomics. 2025 Oct 1;26:879. doi: 10.1186/s12864-025-11940-z (PMC12486977; doi:10.1186/s12864-025-11940-z)
Supplement: Supplementary file 2 — Supplementary Material 2. [file 12864_2025_11940_MOESM2_ESM.docx]

Provision of the genomic inflation factor $\boldsymbol{\lambda}_{\boldsymbol{GC}}$ for each GWAS method

**Table 4:** Genomic inflation factor for different GWAS methods analyzing the variance of the deviation between observed and predicted daily milk yield $v_{d}$ in primiparous (P) and multiparous (M) cows

| **Breed** | **Lactation** | **MLMA** | **MLMA Loco** | **MLMA Loco PC_Genome_** | **MLMA Loco PC_Chromosome_** |
| --- | --- | --- | --- | --- | --- |
| German Holstein | P | 0.811 | 2.257 | 1.621 | 0.503 |
| German Holstein | M | 0.805 | 2.798 | 1.908 | 0.548 |
| German Fleckvieh | P | 0.883 | 1.411 | 1.197 | 0.634 |
| German Fleckvieh | M | 0.840 | 1.703 | 1.374 | 0.676 |
| German Brown Swiss | P | 0.901 | 1.177 | 0.919 | 0.404 |
| German Brown Swiss | M | 0.881 | 1.287 | 1.004 | 0.376 |
| Pooled data set | P | 1.091 | | | |
| Pooled data set | M | 1.255 | | | |
| MLMA – mixed linear model based association analysis  MLMA Loco – MLMA with excluding the genomic relationship matrix of the considered BTA  MLMA Loco PC_Genome_ – MLMA Loco corrected for 20 principal components calculated from the whole genome  MLMA Loco PC_Chromosome_ – MLMA Loco corrected for 20 principal components calculated from the considered chromosome | | | | | |

**Table 5:** Genomic inflation factor for different GWAS methods analyzing the variance of the deviation between observed and predicted daily milk yield $v_{r}$ in primiparous (P) and multiparous (M) cows

| **Breed** | **Lactation** | **MLMA** | **MLMA Loco** | **MLMA Loco PC_Genome_** | **MLMA Loco PC_Chromosome_** |
| --- | --- | --- | --- | --- | --- |
| German Holstein | P | 0.811 | 2.517 | 1.664 | 0.490 |
| German Holstein | M | 0.810 | 2.876 | 1.888 | 0.526 |
| German Fleckvieh | P | 0.864 | 1.345 | 1.155 | 0.628 |
| German Fleckvieh | M | 0.833 | 1.687 | 1.373 | 0.664 |
| German Brown Swiss | P | 0.902 | 1.194 | 0.889 | 0.391 |
| German Brown Swiss | M | 0.825 | 1.284 | 0.971 | 0.385 |
| Pooled data set | P | 1.090 | | | |
| Pooled data set | M | 1.278 | | | |
| MLMA – mixed linear model based association analysis  MLMA Loco – MLMA with excluding the genomic relationship matrix of the considered BTA  MLMA Loco PC_Genome_ – MLMA Loco corrected for 20 principal components calculated from the whole genome  MLMA Loco PC_Chromosome_ – MLMA Loco corrected for 20 principal components calculated from the considered chromosome | | | | | |

**Table 6:** Genomic inflation factor for different GWAS methods analyzing the variance of the deviation between observed and predicted daily milk yield $r_{Auto}$ in primiparous (P) and multiparous (M) cows

| **Breed** | **Lactation** | **MLMA** | **MLMA Loco** | **MLMA Loco PC_Genome_** | **MLMA Loco PC_Chromosome_** |
| --- | --- | --- | --- | --- | --- |
| German Holstein | P | 0.842 | 2.409 | 1.710 | 0.538 |
| German Holstein | M | 0.823 | 2.649 | 1.847 | 0.559 |
| German Fleckvieh | P | 0.858 | 1.324 | 1.117 | 0.612 |
| German Fleckvieh | M | 0.828 | 1.702 | 1.353 | 0.659 |
| German Brown Swiss | P | 0.891 | 1.132 | 0.852 | 0.382 |
| German Brown Swiss | M | 0.853 | 1.270 | 0.997 | 0.413 |
| Pooled data set | P | 1.049 | | | |
| Pooled data set | M | 1.266 | | | |
| MLMA – mixed linear model based association analysis  MLMA Loco – MLMA with excluding the genomic relationship matrix of the considered BTA  MLMA Loco PC_Genome_ – MLMA Loco corrected for 20 principal components calculated from the whole genome  MLMA Loco PC_Chromosome_ – MLMA Loco corrected for 20 principal components calculated from the considered chromosome | | | | | |
