# Supplementary material for: Mapping genes for resilient dairy cows by means of across-breed genome-wide association analysis
Source: BMC Genomics. 2025 Oct 1;26:879. doi: 10.1186/s12864-025-11940-z (PMC12486977; doi:10.1186/s12864-025-11940-z)
Supplement: Supplementary file 3 — Supplementary Material 3. [file 12864_2025_11940_MOESM3_ESM.docx]

**Provision of information of genome-wide significant SNPs and correlated QTL**

**Table 7:** Name and Base-pair Position of genomewide significant SNPs ($p \leq\frac{0.05}{Number of SNPs}$) for the resilience indicators variance ($v_{d}$) and autocorrelation ($r_{Auto}$) of deviations between observed and predicted absolute daily milk yield, variance of relative daily milk yield ($v_{r}$) and a selection index resilience computed from the resilience indicator traits ($SI$) analyzed in across-breeds in first lactation ($P$) and first and higher lactations ($M$)

| **Trait**  **Lactation** | **Marker Name** | **Chromosome** | **Base-pair Position** |
| --- | --- | --- | --- |
| $\boldsymbol{v}_{\boldsymbol{d}}$  P | BovineHD1400000204 | 14 | 487527 |
|  | BovineHD1400000206 | 14 | 494621 |
|  | BovineHD1400000216 | 14 | 550784 |
|  | BovineHD1400000281 | 14 | 854163 |
| $\boldsymbol{v}_{\boldsymbol{d}}$  M | ARS-BFGL-NGS-107379 | 14 | 862483 |
|  | BovineHD1400000188 | 14 | 403312 |
|  | BovineHD1400000199 | 14 | 452477 |
|  | BovineHD1400000200 | 14 | 454838 |
|  | BovineHD1400000204 | 14 | 487527 |
|  | BovineHD1400000206 | 14 | 494621 |
|  | BovineHD1400000216 | 14 | 550784 |
|  | BovineHD1400000275 | 14 | 827586 |
|  | BovineHD1400000281 | 14 | 854163 |
|  | BovineHD1400000283 | 14 | 865655 |
|  | BovineHD1400000286 | 14 | 876529 |
|  | BovineHD1400000287 | 14 | 883732 |
|  | BovineHD1400000324 | 14 | 1063078 |
| $\boldsymbol{v}_{\boldsymbol{r}}$  M | ARS-BFGL-NGS-72413 | 5 | 82061755 |
|  | ARS-BFGL-NGS-80785 | 5 | 75143850 |
|  | BovineHD0500021484 | 5 | 75039090 |
|  | BovineHD0500021485 | 5 | 75048931 |
|  | BovineHD0500021495 | 5 | 75088299 |
|  | BovineHD0500021496 | 5 | 75094522 |
|  | BovineHD0500021497 | 5 | 75098897 |
|  | BovineHD0500021498 | 5 | 75100664 |
|  | BovineHD0500021499 | 5 | 75109095 |
|  | BovineHD0500021500 | 5 | 75111711 |
|  | BovineHD0500021501 | 5 | 75115270 |
|  | BovineHD0500021502 | 5 | 75117210 |
|  | BovineHD0500021505 | 5 | 75129323 |
|  | BovineHD0500021508 | 5 | 75137447 |
|  | BovineHD0500022023 | 5 | 77146567 |
|  | BovineHD0500022699 | 5 | 79710128 |
|  | BovineHD0500023284 | 5 | 82057231 |
|  | BovineHD0500023286 | 5 | 82059684 |
|  | BovineHD0500023287 | 5 | 82060341 |
|  | BovineHD0500023289 | 5 | 82062576 |
|  | BovineHD0500023290 | 5 | 82064350 |
|  | BovineHD0500023291 | 5 | 82064946 |
|  | BovineHD0500023292 | 5 | 82065496 |
|  | BovineHD0500023293 | 5 | 82067669 |
|  | BovineHD0500023295 | 5 | 82077397 |
|  | BovineHD0500023297 | 5 | 82089704 |
|  | BovineHD0500023298 | 5 | 82094211 |
|  | BovineHD0500024876 | 5 | 87262266 |
|  | BovineHD0500024896 | 5 | 87377813 |
|  | BovineHD0500025559 | 5 | 89572280 |
|  | BovineHD0500025685 | 5 | 90038070 |
|  | BovineHD4100003858 | 5 | 75105523 |
|  | BovineHD4100003859 | 5 | 75124656 |
|  | BovineHD4100003886 | 5 | 82066707 |
|  | BovineHD4100003887 | 5 | 82070754 |
|  | BovineHD4100003888 | 5 | 82085293 |
|  | Hapmap40676-BTA-121691 | 5 | 75086818 |
|  | Hapmap50084-BTA-74042 | 5 | 75043240 |
| $\boldsymbol{r}_{\boldsymbol{Auto}}$  M | BTA-114769-no-rs | 8 | 36910551 |
|  | BovineHD0200016228 | 2 | 56978160 |
|  | BovineHD0800011049 | 8 | 36865281 |

**Table 8:** Overview of traits influenced by with genome-wide significant SNPs ($p \leq\frac{0.05}{Number of SNPs}$) for the resilience indicators variance ($v_{d}$) and autocorrelation ($r_{Auto}$) of deviations between observed and predicted absolute daily milk yield, variance of relative daily milk yield ($v_{r}$) and a selection index resilience computed from the resilience indicator traits ($SI$) analyzed in across-breeds in first lactation ($P$) and first and higher lactations ($M$) correlated QTL

| **Trait influenced by associated QTL** | $\boldsymbol{v}_{\boldsymbol{d}}$  P | $\boldsymbol{v}_{\boldsymbol{d}}$  M | $\boldsymbol{v}_{\boldsymbol{r}}$  M | $\boldsymbol{r}_{\boldsymbol{Auto}}$  M |
| --- | --- | --- | --- | --- |
| Body weight |  |  | X |  |
| Bone quality |  |  | X |  |
| Bovine tuberculosis susceptibility |  | X |  | X |
| Calving Interval |  |  |  | X |
| Clinical mastitis | X | X |  |  |
| Conception rate | X | X | X |  |
| Connective tissue amount |  |  | X |  |
| Curd firmness |  | X |  |  |
| Curd solids yield |  | X |  |  |
| Curd yield |  | X |  |  |
| Dystocia |  |  | X |  |
| First service conception |  |  |  | X |
| Heat tolerance | X | X |  | X |
| Ketosis | X | X |  |  |
| Lean meat yield | X | X |  |  |
| Lifetime profit index | X | X |  |  |
| M. paratuberculosis susceptibility |  |  | X |  |
| Marbling score | X | X |  |  |
| Metabolic body weight |  | X |  |  |
| Milk alpha-lactalbumin percentage | X | X |  |  |
| Milk alpha-S1-casein percentage |  | X |  |  |
| Milk beta-lactoglobulin percentage |  | X |  |  |
| Milk butyric acid content | X | X |  |  |
| Milk C14 index | X | X |  |  |
| Milk C16 index | X | X |  |  |
| Milk C18 index | X | X |  |  |
| Milk calcium content | X | X |  |  |
| Milk capric acid content | X | X |  |  |
| Milk caprylic acid content | X | X |  |  |
| Milk casein percentage | X | X |  |  |
| Milk cholesterol content | X | X |  |  |
| Milk citrate count |  | X |  |  |
| Milk conjugated linoleic acid content | X | X |  |  |
| Milk copper content | X | X |  |  |
| Milk fat percentage | X | X | X |  |
| Milk fat yield | X | X | X |  |
| Milk fat-to-protein ratio | X | X |  |  |
| Milk kappa-casein content | X | X |  |  |
| Milk kappa-casein percentage | X | X |  |  |
| Milk lactose yield | X | X |  |  |
| Milk linoleic acid content | X | X |  |  |
| Milk linolenic acid content | X | X |  |  |
| Milk monounsaturated fatty acid content |  | X |  |  |
| Milk myristic acid content | X | X |  |  |
| Milk myristoleic acid content | X | X |  |  |
| Milk oleic acid content | X | X |  |  |
| Milk palmitic acid content | X | X |  |  |
| Milk palmitoleic acid content | X | X |  |  |
| Milk pentadecylic acid content | X | X |  |  |
| Milk phosphorus content |  | X |  |  |
| Milk polyunsaturated fatty acid content |  | X |  |  |
| Milk potassium content | X | X |  |  |
| Milk protein content | X | X |  |  |
| Milk protein percentage | X | X |  |  |
| Milk protein yield | X | X |  |  |
| Milk riboflavin content | X | X |  |  |
| Milk saturated fatty acid content | X | X |  |  |
| Milk stearic acid content | X | X |  |  |
| Milk tridecylic acid content |  | X |  |  |
| Milk unsaturated fatty acid content |  | X |  |  |
| Milk urea nitrogen content | X | X |  |  |
| Milk yield | X | X | X | X |
| Milk zinc content | X | X |  |  |
| Multiple birth |  |  | X |  |
| Pregnancy rate | X | X | X |  |
| Shear force |  |  | X |  |
| Somatic cell count | X | X |  |  |
| Somatic cell score | X | X |  |  |
| Subcutaneous fat thickness |  | X |  |  |
| Udder attachment | X | X |  |  |
| Udder cleft | X | X |  |  |
