# Supplementary material for: Mapping genes for resilient dairy cows by means of across-breed genome-wide association analysis
Source: BMC Genomics. 2025 Oct 1;26:879. doi: 10.1186/s12864-025-11940-z (PMC12486977; doi:10.1186/s12864-025-11940-z)
Supplement: Supplementary file 4 — Supplementary Material 4. [file 12864_2025_11940_MOESM4_ESM.docx]

Manhattan-Plots and QQ-Plots of each breed and lactation group

***Figure 7:*** *Manhattan-Plot of p-values from GWAS in German Brown Swiss for the resilience indicator trait* $v_{d}$*, which is the variance of deviation between observed and predicted absolute daily milk yield in first and higher lactations*


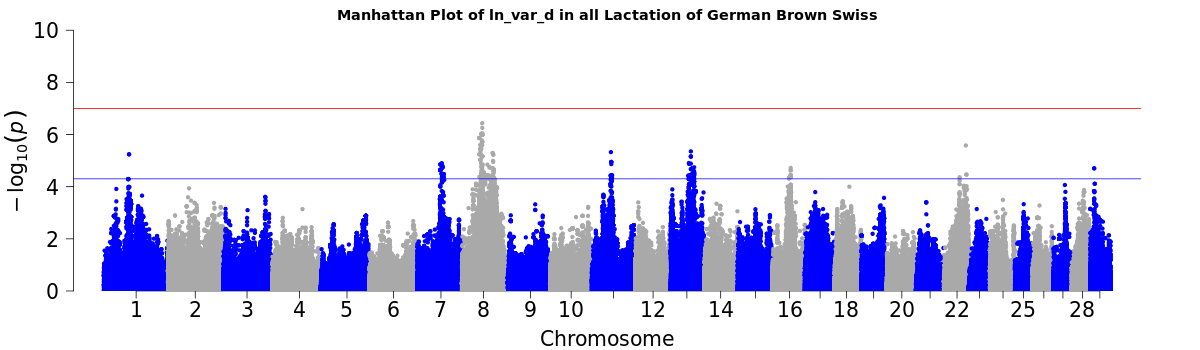

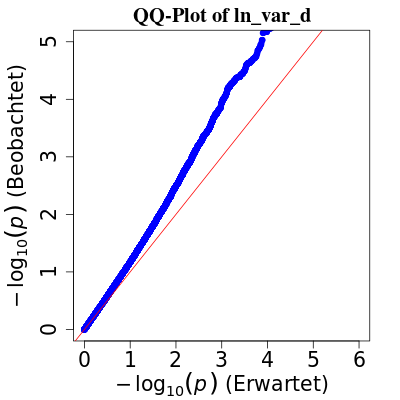


$\mathbf{v}_{\mathbf{d}}$**,**

***Figure 8:*** *Q-Q-Plot of p-values from GWAS in German Brown Swiss for the resilience indicator trait* $v_{d}$*, which is the variance of deviation between observed and predicted absolute daily milk yield in first and higher lactations*


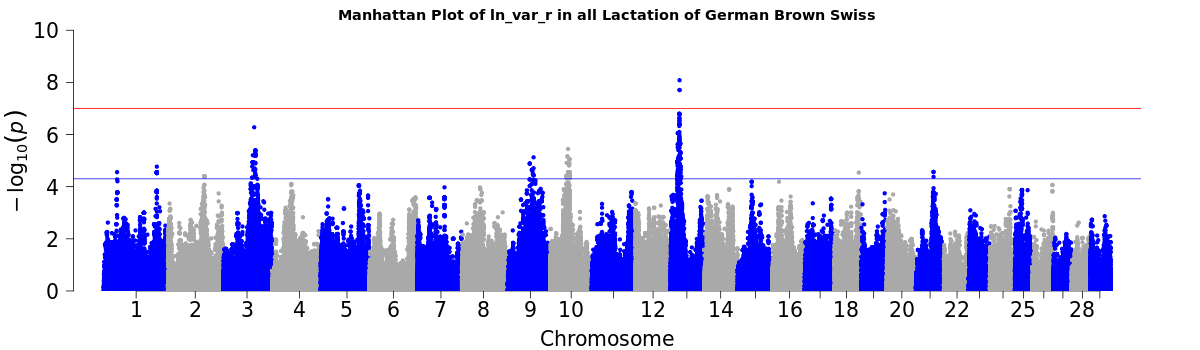


***Figure 9:*** *Manhattan-Plot of p-values from GWAS in German Brown Swiss for the resilience indicator trait* $v_{r}$*, which is the variance of relative daily milk yield in first and higher lactations*


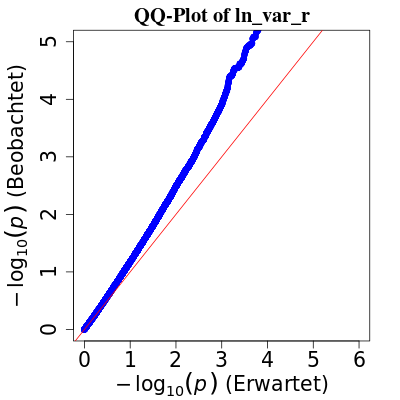


$\mathbf{v}_{\mathbf{r}}$**,**

***Figure 10:*** *Q-Q-Plot of p-values from GWAS in German Brown Swiss for the resilience indicator trait* $v_{r}$*, which is the variance of deviation between observed and predicted absolute daily milk yield in first and higher lactations*


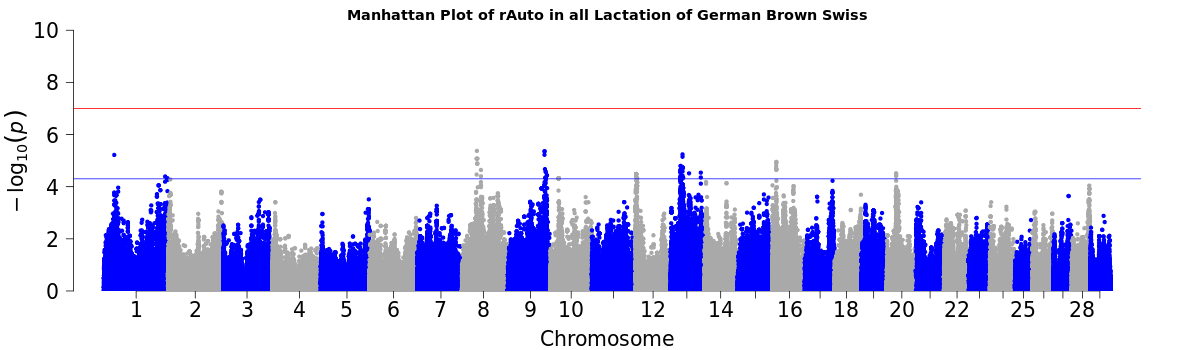


***Figure 11:*** *Manhattan-Plot of p-values from GWAS in German Brown Swiss for resilience indicator trait* $r_{Auto}$*, which is the autocorrelation of deviation between observed and predicted daily milk yield, in first and higher lactations*


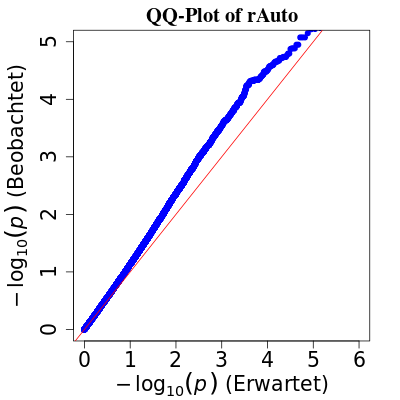


$\mathbf{r}_{\mathbf{Auto}}$**,**

***Figure 12:*** *Q-Q-Plot of p-values from GWAS in German Brown Swiss for the resilience indicator trait* $r_{Auto}$*, which is the variance of deviation between observed and predicted absolute daily milk yield in first and higher lactations*

***Figure 13:*** *Manhattan-Plot of p-values from GWAS in German Brown Swiss for the resilience selection index* $SI$ *computed from resilience indicator traits calculated from resilience indicator traits in first and higher lactations*


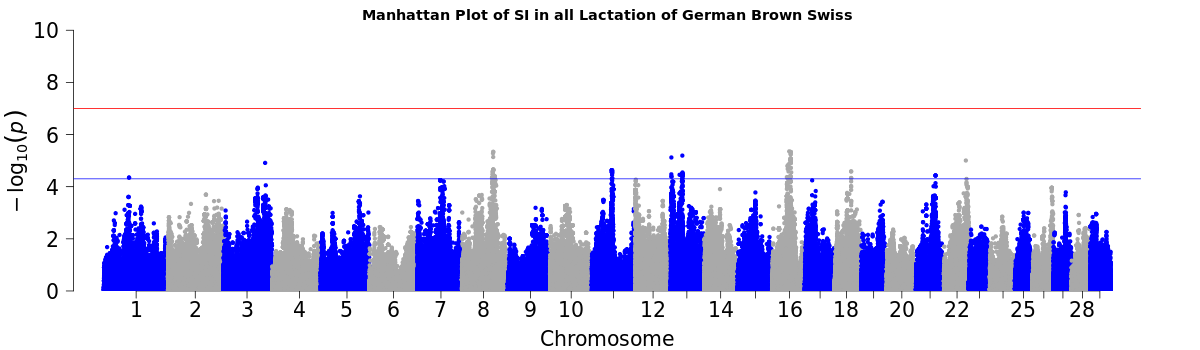

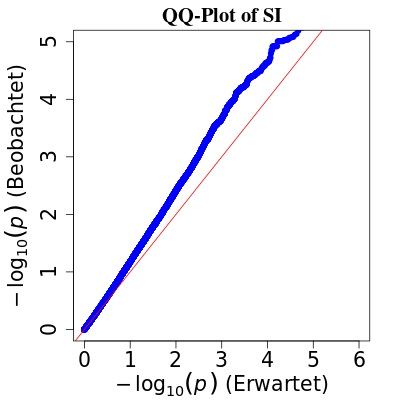


$\mathbf{SI}$**,**

***Figure 14:*** *Q-Q-Plot of p-values from GWAS in German Brown Swiss for the resilience indicator trait* $SI$*, which is the variance of deviation between observed and predicted absolute daily milk yield in first and higher lactations*

***Figure 15:*** *Manhattan-Plot of p-values from GWAS in German Brown Swiss for the resilience indicator trait* $v_{d}$*, which is the variance of deviation between observed and predicted absolute daily milk yield in first lactation*


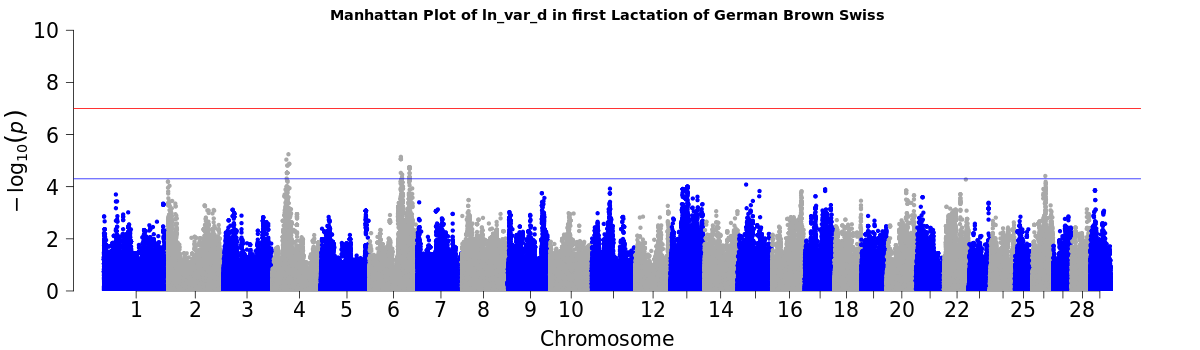

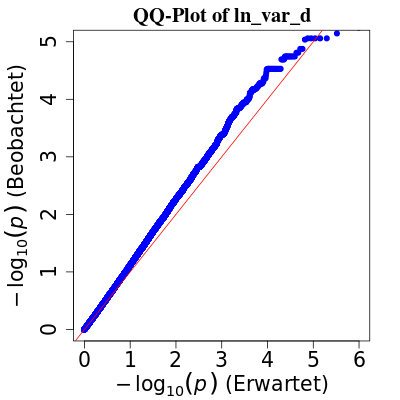


$\mathbf{v}_{\mathbf{d}}$**,**

***Figure 16:*** *Q-Q-Plot of p-values from GWAS in German Brown Swiss for the resilience indicator trait* $v_{d}$*, which is the variance of deviation between observed and predicted absolute daily milk yield in first lactation*


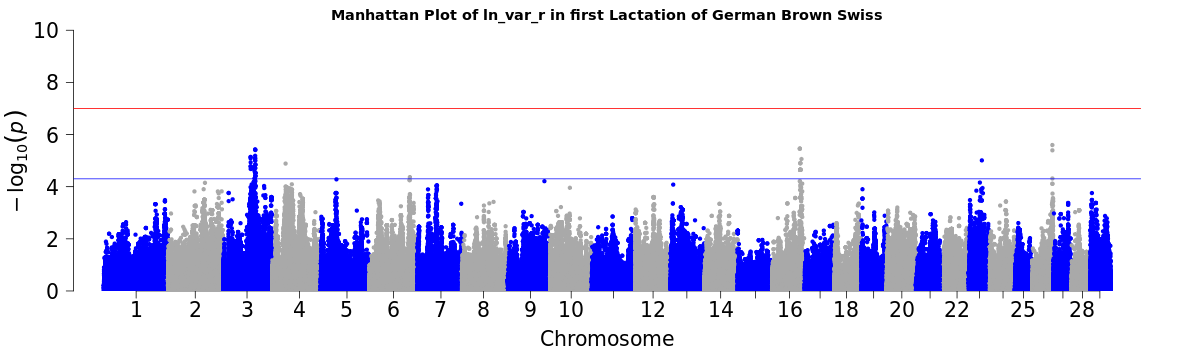


***Figure 17:*** *Manhattan-Plot of p-values from GWAS in German Brown Swiss for the resilience indicator trait* $v_{r}$*, which is the variance of relative daily milk yield in first lactation*


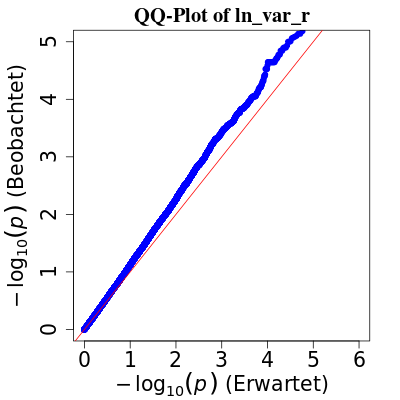


$\mathbf{v}_{\mathbf{r}}$**,**

***Figure 18:*** *Q-Q-Plot of p-values from GWAS in German Brown Swiss for the resilience indicator trait* $v_{r}$*, which is the variance of deviation between observed and predicted absolute daily milk yield in first lactation*

***Figure 19:*** *Manhattan-Plot of p-values from GWAS in German Brown Swiss for resilience indicator trait* $r_{Auto}$*, which is the autocorrelation of deviation between observed and predicted daily milk yield, in first lactation*


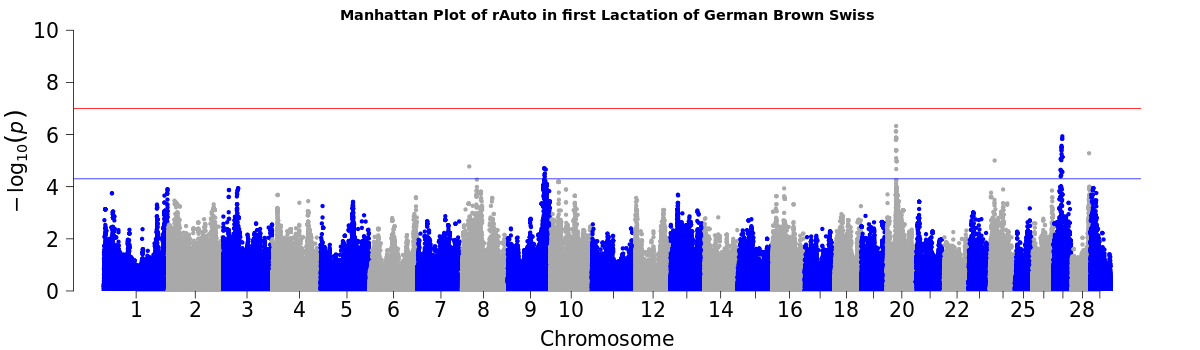

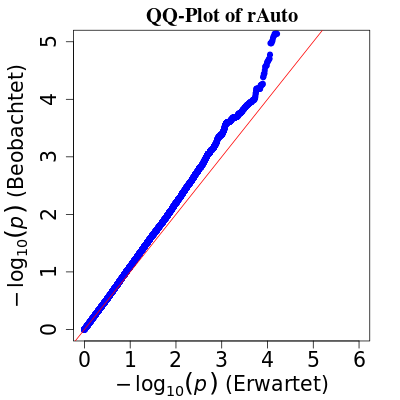


$\mathbf{r}_{\mathbf{Auto}}$**,**

***Figure 20:*** *Q-Q-Plot of p-values from GWAS in German Brown Swiss for the resilience indicator trait* $r_{Auto}$*, which is the variance of deviation between observed and predicted absolute daily milk yield in first lactation*

***Figure 21:*** *Manhattan-Plot of p-values from GWAS in German Brown Swiss for the resilience selection index* $SI$ *computed from resilience indicator traits calculated from resilience indicator traits in first lactation*


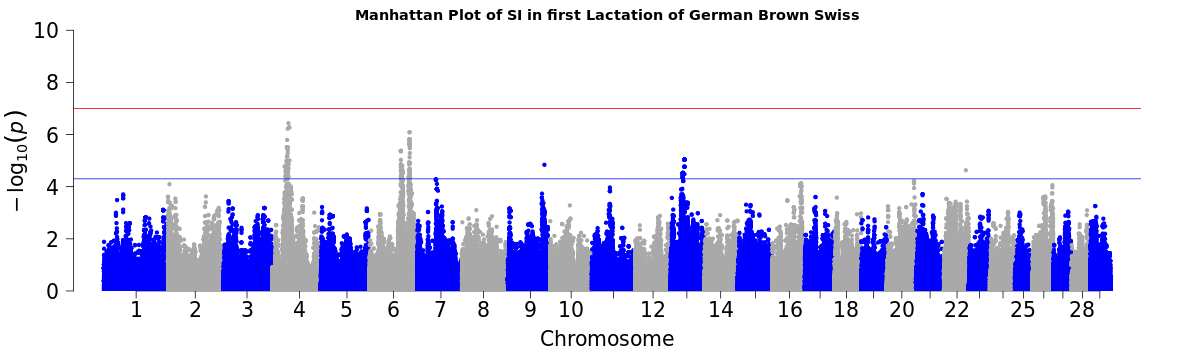

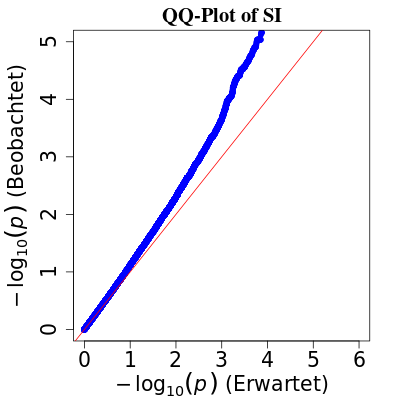


$\mathbf{SI}$**,**

***Figure 22:*** *Q-Q-Plot of p-values from GWAS in German Brown Swiss for the resilience indicator trait* $SI$*, which is the variance of deviation between observed and predicted absolute daily milk yield in first lactation*


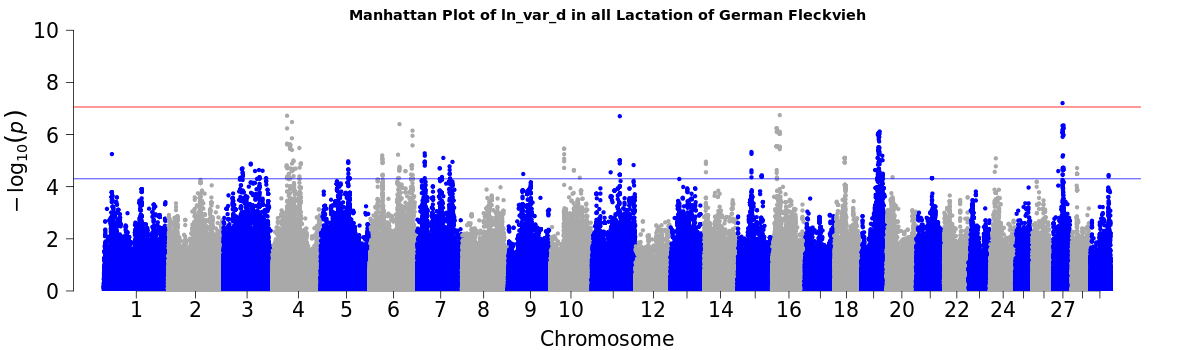


***Figure 23:*** *Manhattan-Plot of p-values from GWAS in Fleckvieh for the resilience indicator trait* $v_{d}$*, which is the variance of deviation between observed and predicted absolute daily milk yield in first and higher lactations*


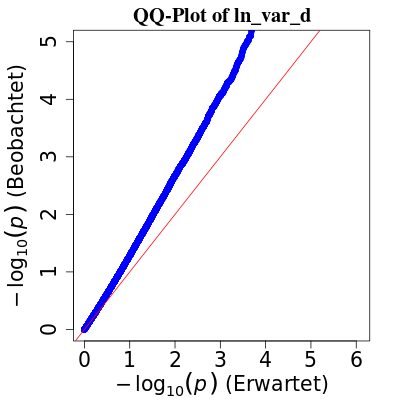


$\mathbf{v}_{\mathbf{d}}$**,**

***Figure 24:*** *Q-Q-Plot of p-values from GWAS in Fleckvieh for the resilience indicator trait* $v_{d}$*, which is the variance of deviation between observed and predicted absolute daily milk yield in first and higher lactations*

***Figure 25:*** *Manhattan-Plot of p-values from GWAS in Fleckvieh for the resilience indicator trait* $v_{r}$*, which is the variance of relative daily milk yield in first and higher lactations*


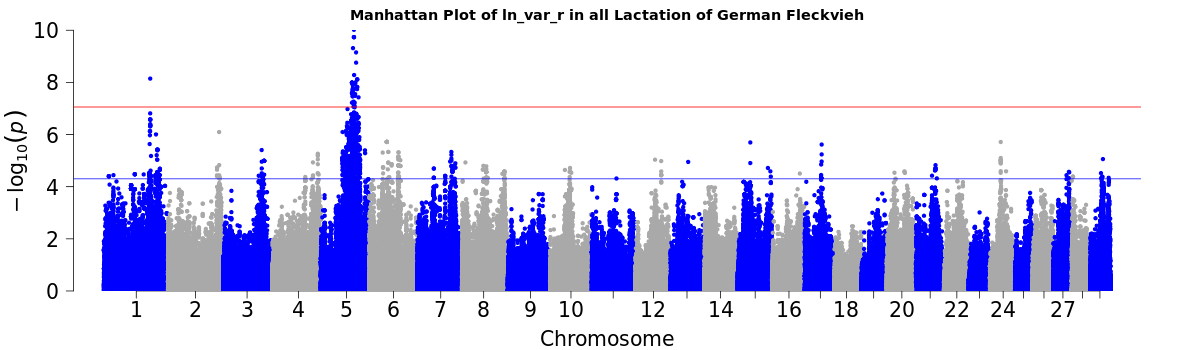

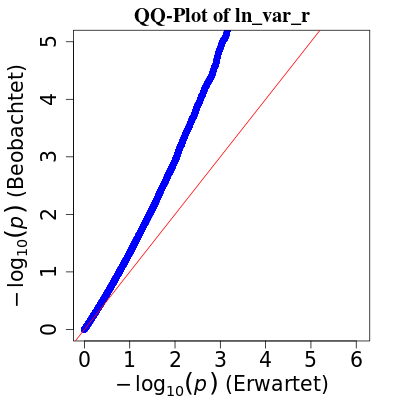


$\mathbf{v}_{\boldsymbol{r}}$**,**

***Figure 26:*** *Q-Q-Plot of p-values from GWAS in Fleckvieh for the resilience indicator trait* $v_{r}$*, which is the variance of deviation between observed and predicted absolute daily milk yield in first and higher lactations*

***Figure 27:*** *Manhattan-Plot of p-values from GWAS in Fleckvieh for resilience indicator trait* $r_{Auto}$*, which is the autocorrelation of deviation between observed and predicted daily milk yield, in first and higher lactations*


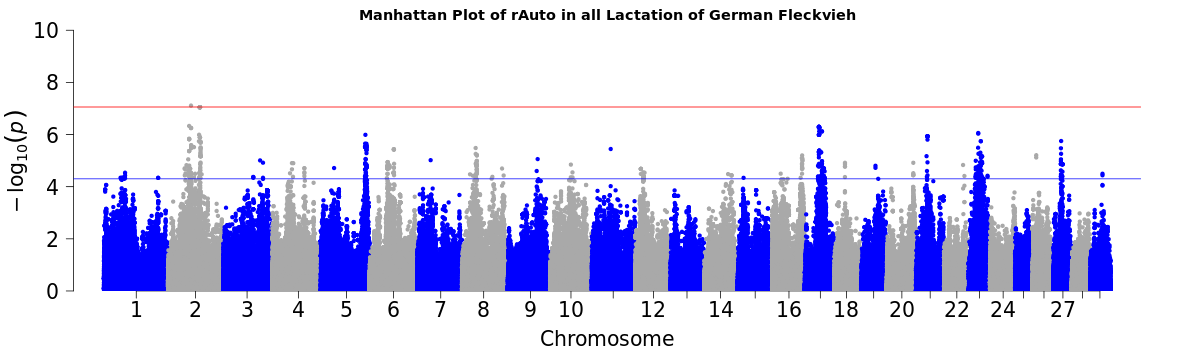

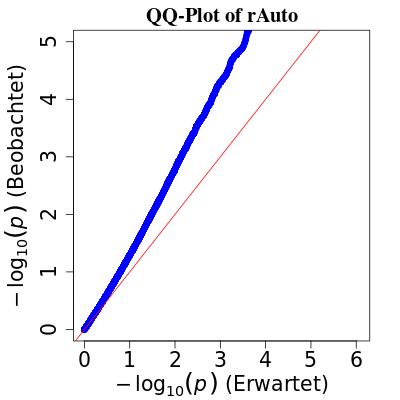


$\mathbf{r}_{\boldsymbol{Auto}}$**,**

***Figure 28:*** *Q-Q-Plot of p-values from GWAS in Fleckvieh for the resilience indicator trait* $r_{Auto}$*, which is the variance of deviation between observed and predicted absolute daily milk yield in first and higher lactations*


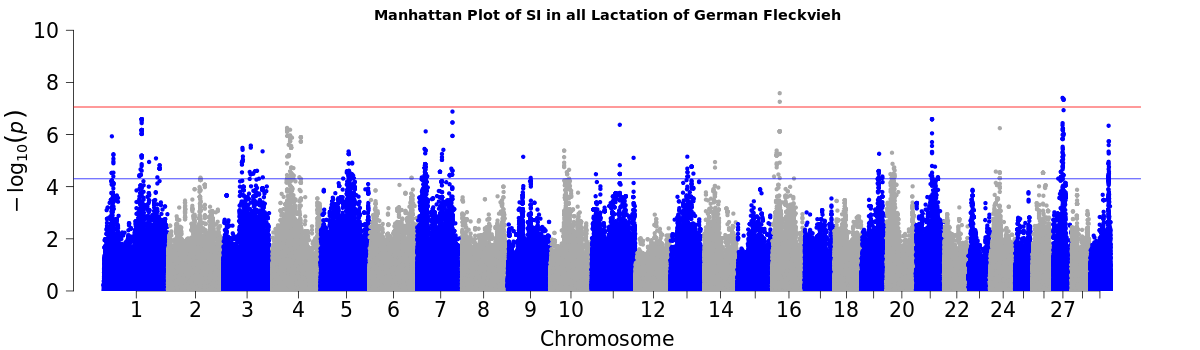


***Figure 29:*** *Manhattan-Plot of p-values from GWAS in Fleckvieh for the resilience selection index* $SI$ *computed from resilience indicator traits calculated from resilience indicator traits in first and higher lactations*


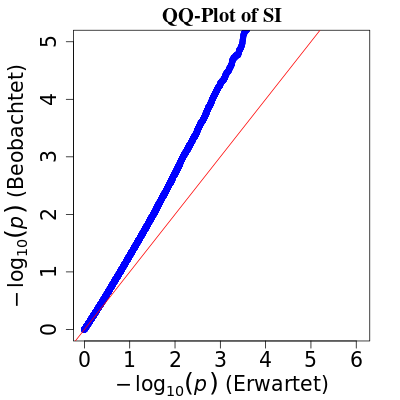

$$\mathbf{SI}$$

**,**

***Figure 30:*** *Q-Q-Plot of p-values from GWAS in Fleckvieh for the resilience indicator trait* $SI$*, which is the variance of deviation between observed and predicted absolute daily milk yield in first and higher lactations*


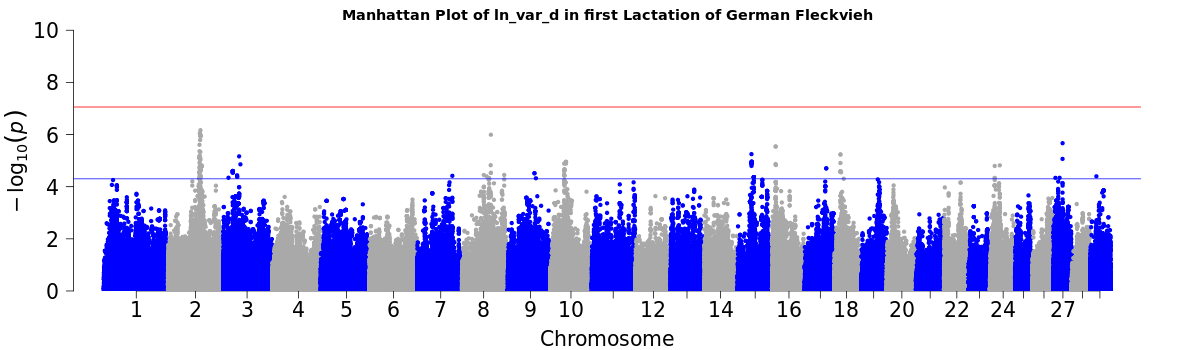


***Figure 31:*** *Manhattan-Plot of p-values from GWAS in Fleckvieh for the resilience indicator trait* $v_{d}$*, which is the variance of deviation between observed and predicted absolute daily milk yield in first lactation*


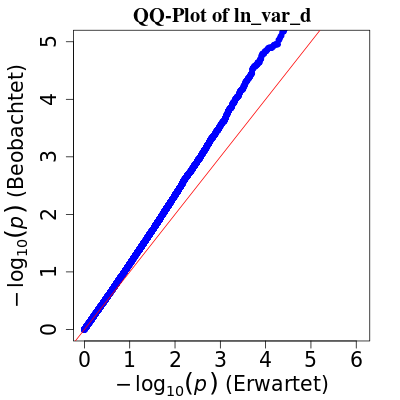


$\mathbf{v}_{\boldsymbol{d}}$**,**

***Figure 32:*** *Q-Q-Plot of p-values from GWAS in Fleckvieh for the resilience indicator trait* $v_{d}$*, which is the variance of deviation between observed and predicted absolute daily milk yield in first lactations*

***Figure 33:*** *Manhattan-Plot of p-values from GWAS in Fleckvieh for the resilience indicator trait* $v_{r}$*, which is the variance of relative daily milk yield in first lactation*


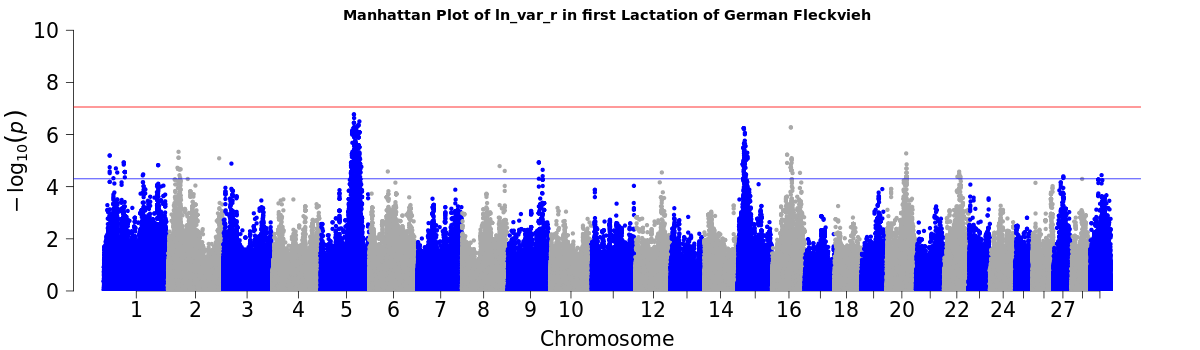

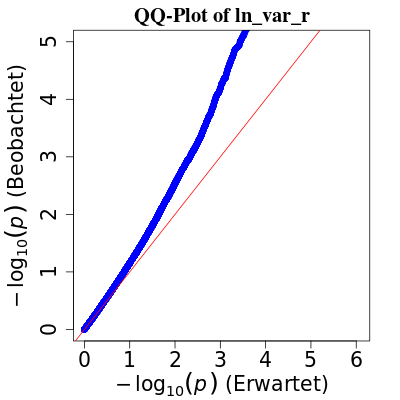


$\mathbf{v}_{\boldsymbol{r}}$**,**

***Figure 34:*** *Q-Q-Plot of p-values from GWAS in Fleckvieh for the resilience indicator trait* $v_{r}$*, which is the variance of deviation between observed and predicted absolute daily milk yield in first lactations*

***Figure 35:*** *Manhattan-Plot of p-values from GWAS in Fleckvieh for resilience indicator trait* $r_{Auto}$*, which is the autocorrelation of deviation between observed and predicted daily milk yield, in first lactation*


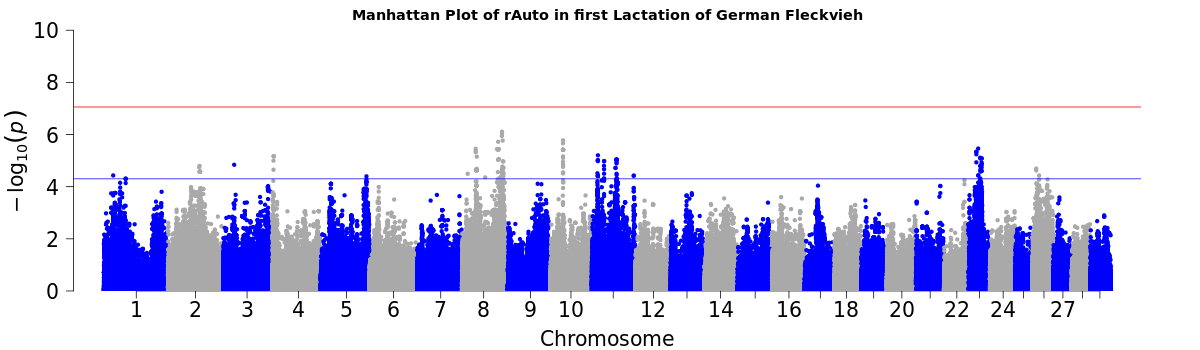

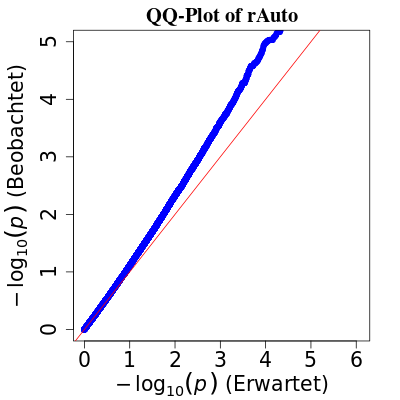


$\mathbf{r}_{\boldsymbol{Auto}}$**,**

***Figure 36:*** *Q-Q-Plot of p-values from GWAS in Fleckvieh for the resilience indicator trait* $r_{Auto}$*, which is the variance of deviation between observed and predicted absolute daily milk yield in first lactations*

***Figure 37:*** *Manhattan-Plot of p-values from GWAS in Fleckvieh for the resilience selection index* $SI$ *computed from resilience indicator traits calculated from resilience indicator traits in first lactation*


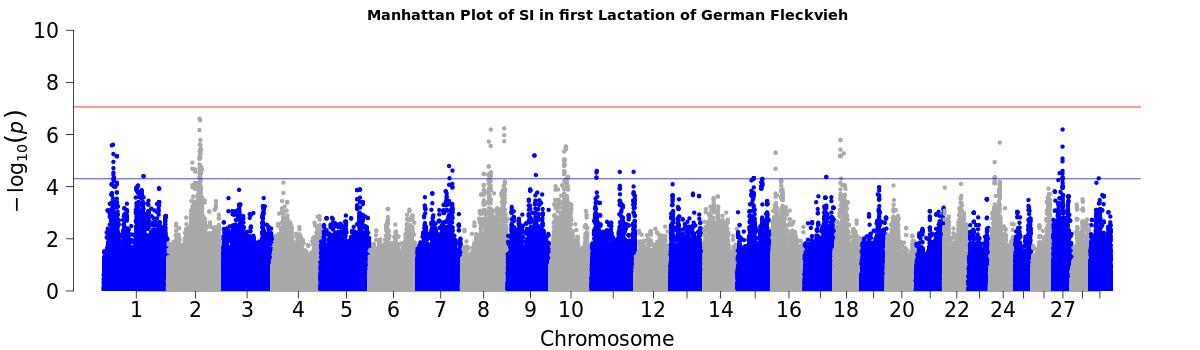

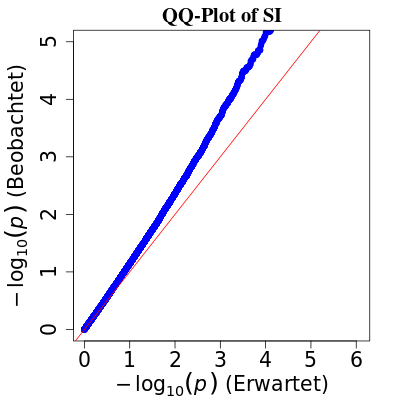


$\mathbf{SI}$**,**

***Figure 38:*** *Q-Q-Plot of p-values from GWAS in Fleckvieh for the resilience indicator trait* $SI$*, which is the variance of deviation between observed and predicted absolute daily milk yield in first lactations*


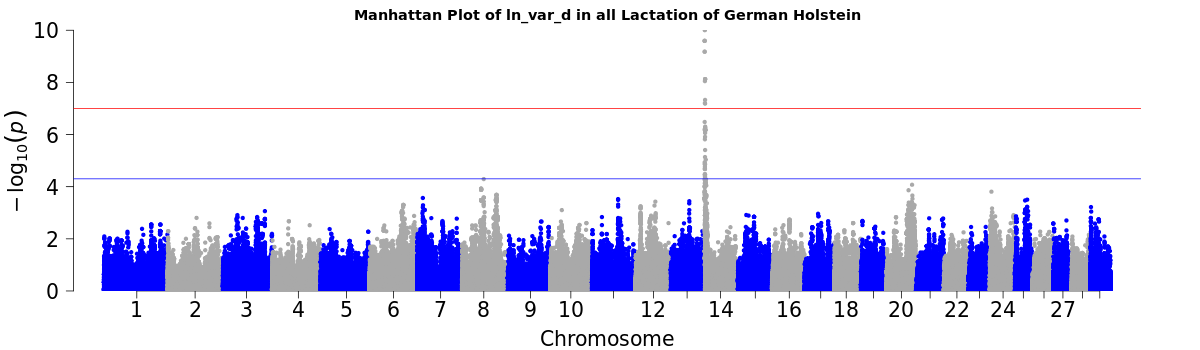


***Figure 39:*** *Manhattan-Plot of p-values from GWAS in German Holstein for the resilience indicator trait* $v_{d}$*, which is the variance of deviation between observed and predicted absolute daily milk yield in first and higher lactations*


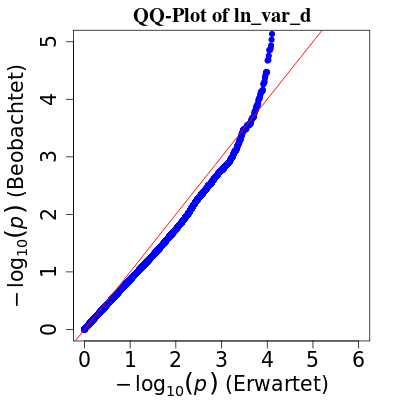


$\mathbf{v}_{\boldsymbol{d}}$**,**

***Figure 40:*** *Q-Q-Plot of p-values from GWAS in German Holstein for the resilience indicator trait* $v_{d}$*, which is the variance of deviation between observed and predicted absolute daily milk yield in first and higher lactations*


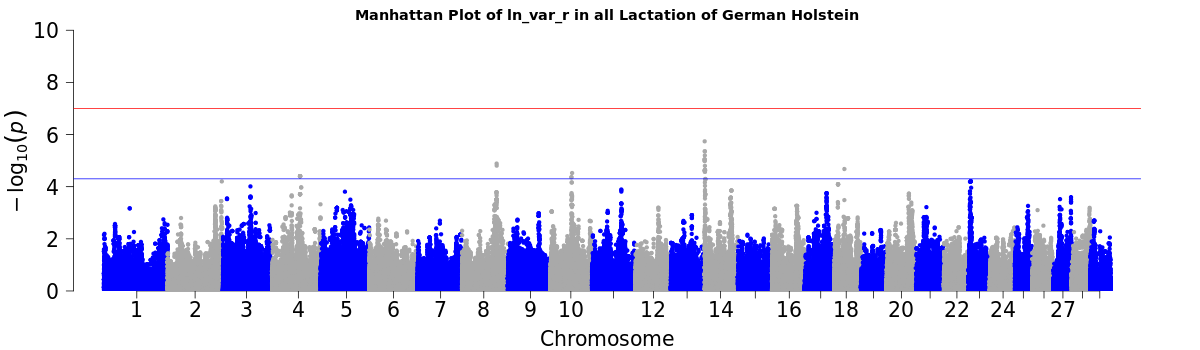


***Figure 41:*** *Manhattan-Plot of p-values from GWAS in German Holstein for the resilience indicator trait* $v_{r}$*, which is the variance of relative daily milk yield in first and higher lactations*


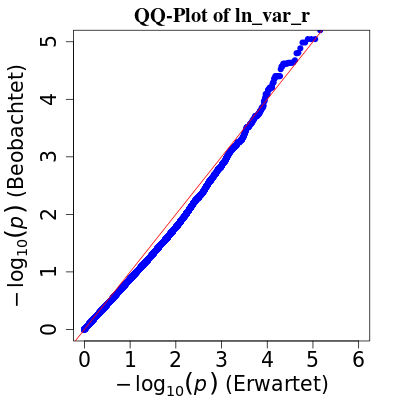


$\mathbf{v}_{\boldsymbol{r}}$**,**

***Figure 42:*** *Q-Q-Plot of p-values from GWAS in German Holstein for the resilience indicator trait* $v_{r}$*, which is the variance of deviation between observed and predicted absolute daily milk yield in first and higher lactations*


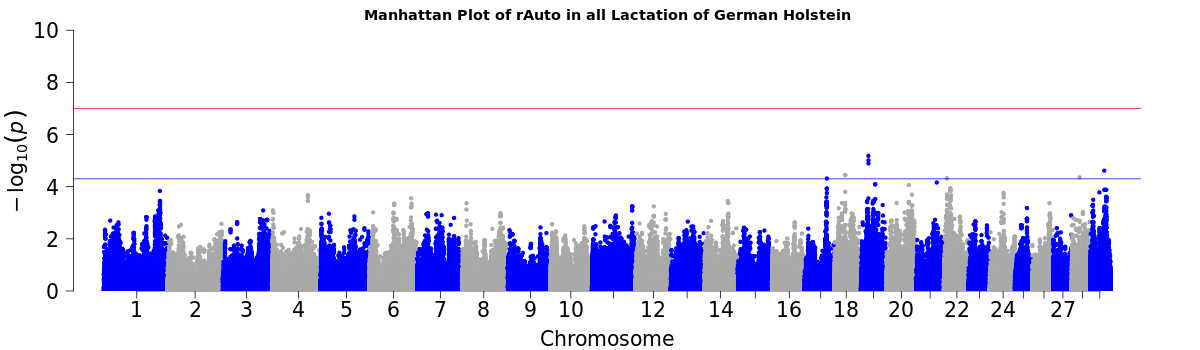


***Figure 43:*** *Manhattan-Plot of p-values from GWAS in German Holstein for resilience indicator trait* $r_{Auto}$*, which is the autocorrelation of deviation between observed and predicted daily milk yield, in first and higher lactations*


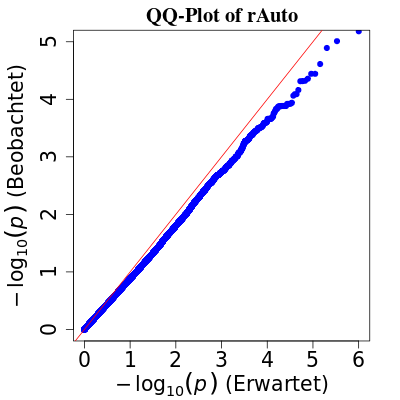


$\mathbf{r}_{\boldsymbol{Auto}}$**,**

***Figure 44:*** *Q-Q-Plot of p-values from GWAS in German Holstein for the resilience indicator trait* $r_{Auto}$*, which is the variance of deviation between observed and predicted absolute daily milk yield in first and higher lactations*

***Figure 45:*** *Manhattan-Plot of p-values from GWAS in German Holstein for the resilience selection index* $SI$ *computed from resilience indicator traits calculated from resilience indicator traits in first and higher lactations*


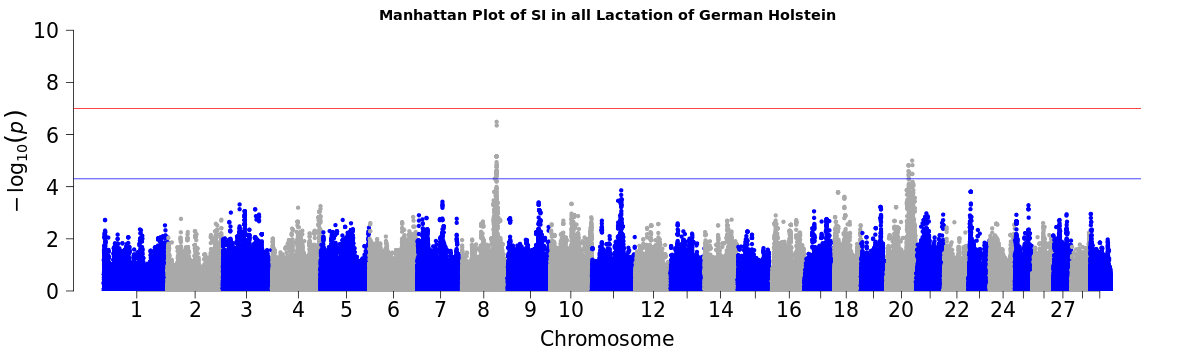

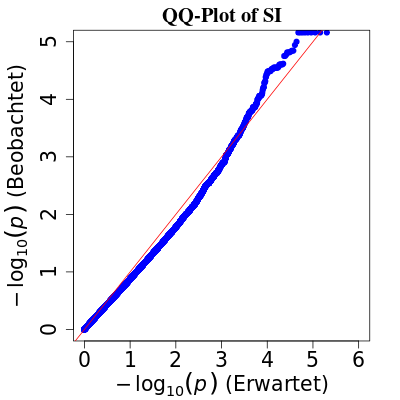


$\mathbf{SI}$**,**

***Figure 46:*** *Q-Q-Plot of p-values from GWAS in German Holstein for the resilience indicator trait* $SI$*, which is the variance of deviation between observed and predicted absolute daily milk yield in first and higher lactations*

***Figure 47:*** *Manhattan-Plot of p-values from GWAS in German Holstein for the resilience indicator trait* $v_{d}$*, which is the variance of deviation between observed and predicted absolute daily milk yield in first lactation*


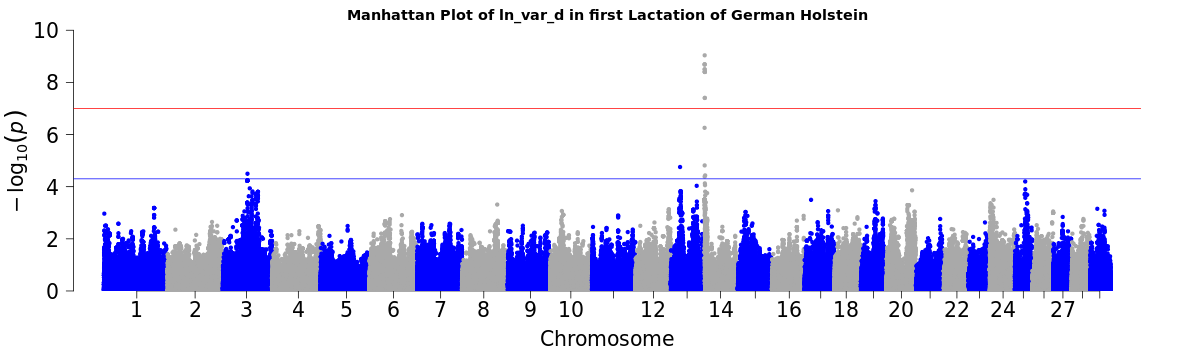

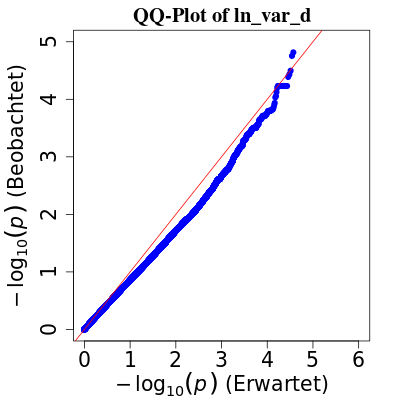


$\mathbf{v}_{\boldsymbol{d}}$**,**

***Figure 48:*** *Q-Q-Plot of p-values from GWAS in German Holstein for the resilience indicator trait* $v_{d}$*, which is the variance of deviation between observed and predicted absolute daily milk yield in first lactation*

***Figure 49:*** *Manhattan-Plot of p-values from GWAS in German Holstein for the resilience indicator trait* $v_{r}$*, which is the variance of relative daily milk yield in first lactation*


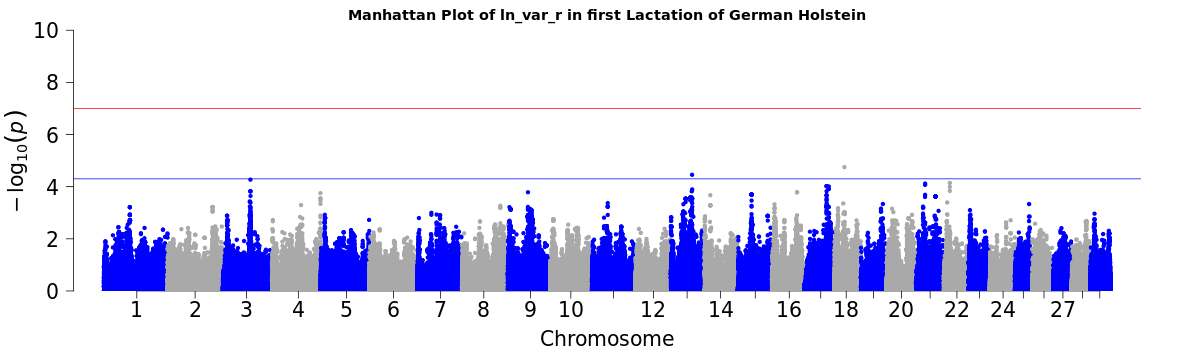

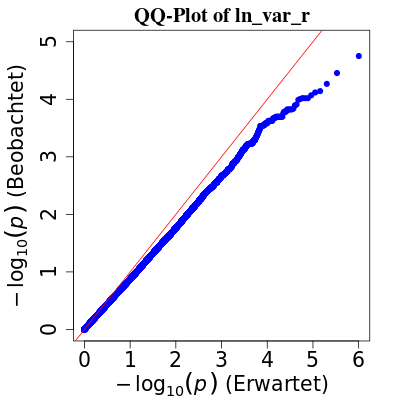


$\mathbf{v}_{\boldsymbol{r}}$**,**

***Figure 50:*** *Q-Q-Plot of p-values from GWAS in German Holstein for the resilience indicator trait* $v_{r}$*, which is the variance of deviation between observed and predicted absolute daily milk yield in first lactation*

***Figure 51:*** *Manhattan-Plot of p-values from GWAS in German Holstein for resilience indicator trait* $r_{Auto}$*, which is the autocorrelation of deviation between observed and predicted daily milk yield, in first lactation*


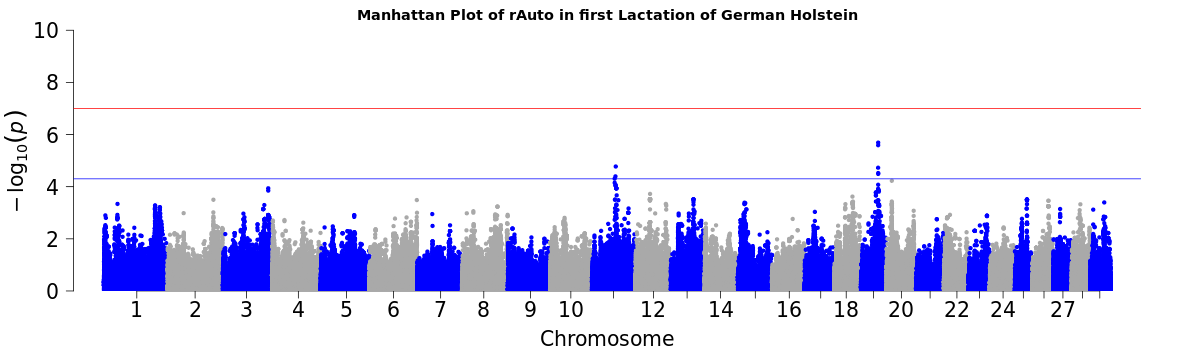

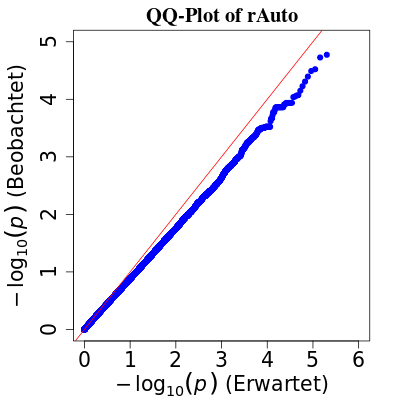


$\mathbf{r}_{\boldsymbol{Auto}}$**,**

***Figure 52:*** *Q-Q-Plot of p-values from GWAS in German Holstein for the resilience indicator trait* $r_{Auto}$*, which is the variance of deviation between observed and predicted absolute daily milk yield in first lactation*

***Figure 53:*** *Manhattan-Plot of p-values from GWAS in German Holstein for the resilience selection index* $SI$ *computed from resilience indicator traits calculated from resilience indicator traits in first lactation*


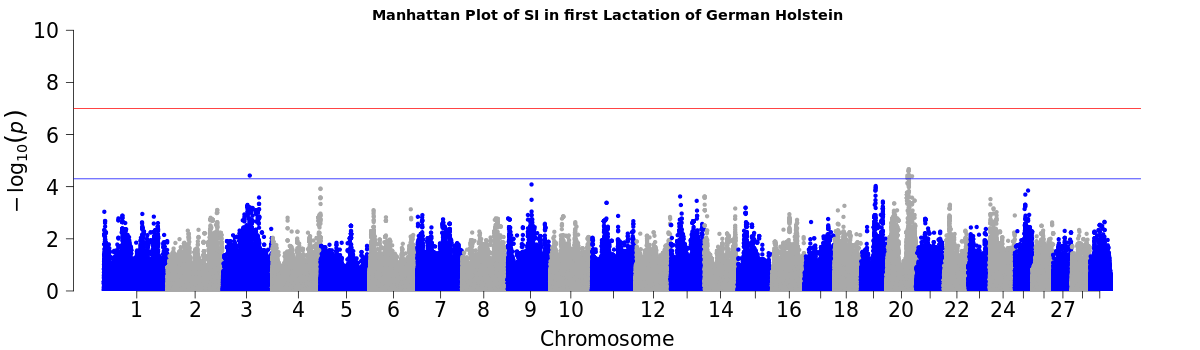

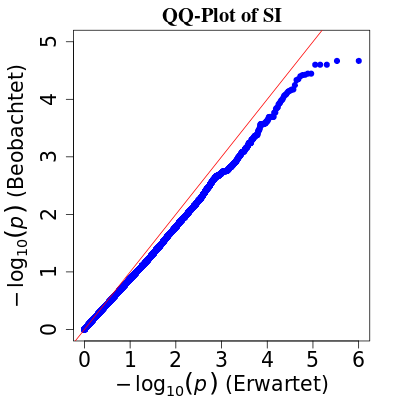


$\mathbf{SI}$**,**

***Figure 54:*** *Q-Q-Plot of p-values from GWAS in German Holstein for the resilience indicator trait* $SI$*, which is the variance of deviation between observed and predicted absolute daily milk yield in first lactation*
